# Repeatability of ^18^F‐FDG PET radiomic features: A phantom study to explore sensitivity to image reconstruction settings, noise, and delineation method

**DOI:** 10.1002/mp.13322

**Published:** 2018-12-28

**Authors:** Elisabeth Pfaehler, Roelof J. Beukinga, Johan R. de Jong, Riemer H. J. A. Slart, Cornelis H. Slump, Rudi A. J. O. Dierckx, Ronald Boellaard

**Affiliations:** ^1^ Department of Nuclear Medicine and Molecular Imaging Medical Imaging Center University of Groningen University Medical Center Groningen Groningen The Netherlands; ^2^ Department of Biomedical Photonic Imaging University of Twente Enschede The Netherlands; ^3^ MIRA Institute for Biomedical Technology and Technical Medicine University of Twente Enschede The Netherlands; ^4^ Department of Radiology & Nuclear Medicine Amsterdam University Medical Centers Location VUMC Amsterdam The Netherlands

**Keywords:** ^18^F‐FDG PET/CT radiomic features, delineation, image reconstruction settings

## Abstract

**Background:**

^18^F‐fluoro‐2‐deoxy‐D‐Glucose positron emission tomography (^18^F‐FDG PET) radiomics has the potential to guide the clinical decision making in cancer patients, but validation is required before radiomics can be implemented in the clinical setting. The aim of this study was to explore how feature space reduction and repeatability of ^18^F‐FDG PET radiomic features are affected by various sources of variation such as underlying data (e.g., object size and uptake), image reconstruction methods and settings, noise, discretization method, and delineation method.

**Methods:**

The NEMA image quality phantom was scanned with various sphere‐to‐background ratios (SBR), simulating different activity uptakes, including spheres with low uptake, that is, SBR smaller than 1. Furthermore, images of a phantom containing 3D printed inserts reflecting realistic heterogeneity uptake patterns were acquired. Data were reconstructed using various matrix sizes, reconstruction algorithms, and scan durations (noise). For every specific reconstruction and noise level, ten statistically equal replicates were generated. The phantom inserts were delineated using CT and PET‐based segmentation methods. A total of 246 radiomic features was extracted from each image dataset. Images were discretized with a fixed number of 64 bins (FBN) and a fixed bin width (FBW) of 0.25 for the high and a FBW of 0.05 for the low uptake data. In terms of feature reduction, we determined the impact of these factors on the composition of feature clusters, which were defined on the basis of Spearman's correlation matrices. To assess feature repeatability, the intraclass correlation coefficient was calculated over the ten replicates.

**Results:**

In general, larger spheres with high uptake resulted in better repeatability compared to smaller low uptake spheres. In terms of repeatability, features extracted from heterogeneous phantom inserts were comparable to features extracted from bigger high uptake spheres. For example, for an EARL‐compliant reconstruction, larger and smaller high uptake spheres yielded good repeatability for 32% and 30% of the features, while the heterogeneous inserts resulted in 34% repeatable features. For the low uptake spheres, this was the case for 22% and 20% of the features for bigger and smaller spheres, respectively. Images reconstructed with point‐spread‐function (PSF) resulted in the highest repeatability when compared with OSEM or time‐of‐flight, for example, 53%, 30%, and 32% of repeatable features, respectively (for unsmoothed data, discretized with FBN, 300 s scan duration). Reducing image noise (increasing scan duration and smoothing) and using CT‐based segmentation for the low uptake spheres yielded improved repeatability. FBW discretization resulted in higher repeatability than FBN discretization, for example, 89% and 35% of the features, respectively (for the EARL‐compliant reconstruction and larger high uptake spheres).

**Conclusion:**

Feature space reduction and repeatability of ^18^F‐FDG PET radiomic features depended on all studied factors. The high sensitivity of PET radiomic features to image quality suggests that a high level of image acquisition and preprocessing standardization is required to be used as clinical imaging biomarker.

## 1 Introduction

^18^F‐fluoro‐2‐deoxy‐D‐Glucose (^18^F‐FDG) positron emission tomography (PET) has become part of the routine oncological diagnostic workup and has been applied for treatment response monitoring and prognosis due to its ability to noninvasively visualize organs and lesions. Although qualitative visual image assessment remains important for these purposes, it has a limited capability to objectively quantify tracer uptake. The most widely used semi‐quantitative measures are the maximum, mean, and peak standardized uptake value (SUV_max_, SUV_mean_, and SUV_peak_) and morphologically based imaging features, such as the metabolic tumor volume or total lesion glycolysis.^1^, ^2^, ^3^ However, these features ignore the intratumoral ^18^F‐FDG spatial distribution.^4^ The rapidly emerging field of ‘radiomics’ computes a large number of quantitative image features to characterize this intratumoral distribution or other tumor phenotypes such as shape.^5^, ^6^, ^7^

Even though radiomics has the potential to add valuable information to the visual image evaluation in various cancer types,^8^ several challenges need to be addressed before radiomics can safely be implemented in the clinic. One of the key problems with generating a multitude of features is the risk of false‐positive findings due to multiple testing. Moreover, numerous features may represent similar tracer uptake characteristics, and may therefore be correlated and redundant.^9^ As models composed of redundant features may become unstable and difficult to interpret, it is required to reduce the feature space to a degree that is manageable for clinical use without losing important information. However, the identification of nonredundant features is challenging. Possible solutions to reduce the feature space would be the use of principal component analysis or (hierarchical) clustering, based on correlation analysis or distance metrics.^10^ Another challenge facing radiomic features is the establishment of their measurement error (i.e., reproducibility, repeatability, and reliability). Several studies have shown that the majority of the ^18^F‐FDG PET radiomic features are sensitive to numerous sources such as image acquisition, reconstruction protocols, or delineation method.^9^, ^11^, ^12^, ^13^, ^14^, ^15^, ^16^, ^17^, ^18^, ^19^ Our study investigates the relationship of multiple confounding factors, including different activity uptake levels, thereby simulating tracers showing differences in uptake. For this purpose, two phantoms (the NEMA image quality (IQ) phantom and a phantom containing in‐house designed 3D printed inserts simulating realistic heterogeneity uptake) were scanned. Multiple factors were varied in order to investigate the relationships between scanner and underlying data‐dependent factors. In contrast to the most other studies, this study focuses on the impact of underlying data characteristics (contrast), image reconstruction methods and settings, noise, discretization method, and delineation method on specifically the dimensionality reduction as well as repeatability of ^18^F‐FDG PET radiomic features.

## 2 Materials and methods

### 2.A. Phantom Experiments

#### 2.A.1. NEMA image quality phantom

The NEMA NU 2‐2012 IQ phantom was used in this study consisting of a background volume of 9400 ml and six fillable spheres with inner diameters of 10, 13, 17, 22, 28, and 37 mm. The phantom was filled with different ^18^F‐FDG concentrations. Two scans with sphere‐to‐background ratios (SBRs) higher than one (about 10:1, 5:1), and two scans with a SBR lower than one (about 0.5:1, and 0.25:1) were acquired. The spheres were filled with 22.6, 10.87, 1.08, and 0.65 kBq/ml measured with a dosiscalibrator (Veenstra instruments, VDC 2.0.2), while the background was filled with 2.4, 2.26, 2.12, and 2.68 kBq/ml, respectively. All phantom scans were acquired as 70 min list‐mode data on a PET/CT system (Biograph mCT‐40 PET/CT, Siemens, Knoxville, TN, USA). The data were reconstructed to obtain a frame of 30, 60, 120, and 300 s. For every scan duration, nine additional frames were reconstructed such that they contained the same amount of counts, taking into account the decay of the tracer. Each dataset was reconstructed using iterative ordered subset expectation maximization (OSEM) algorithm (3 iterations, 24 subsets) and the vendor provided time‐of‐flight (TOF) iterative reconstruction method (3 iterations, 21 subsets). Furthermore, all scans were reconstructed with and without resolution modeling (or point‐spread‐function (PSF)). The data were reconstructed with an image matrix size of 256 × 256 × 111 and a voxel size of 3.01 × 3.01 × 2 mm. The TOF reconstructions with and without PSF were also obtained with a matrix size of 400 × 400 × 111 leading to a cubic voxel size of 2 mm, as a cubic voxel size is recommended for feature extraction.^8^ A low‐dose CT scan (80 kV, 30 mAs, and 2 mm slice thickness) of the phantom was generated in order to calculate the attenuation map of the PET image. To obtain quantitative PET data, images were corrected for attenuation, scatter, random coincidences, and normalization. Images were smoothed with Gaussian filters of 0, 2, 4, 6, and 8 mm full width at half maximum (FWHM) and were converted to SUV, so that the mean phantom background SUV was equal to 1.^20^

#### 2.A.2. 3D printed phantom inserts

**Figure 1:**
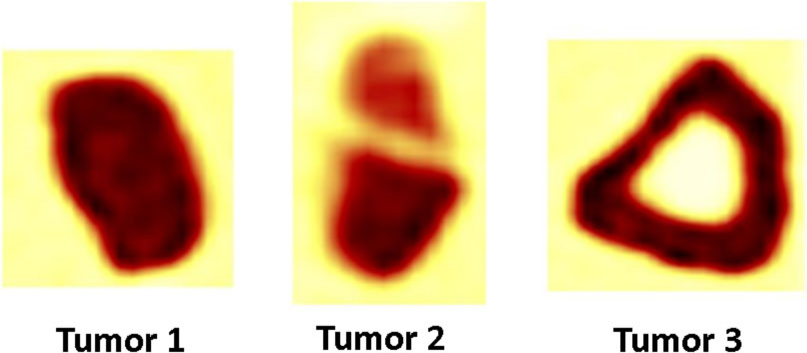
PET images showing results with 3D printed inserts: tumor 1 with homogeneous uptake, tumor 2 with heterogeneity over 2 compartments, and tumor 3 containing a necrotic core (from left to right) [Color figure can be viewed at wileyonlinelibrary.com]

Additionally, a second phantom scan was performed. The spheres of the IQ phantom were replaced by three 3D printed inserts simulating heterogeneous uptake and realistic tumor shapes. The inserts were designed according to Non‐Small‐Cell‐Lung‐Cancer (NSCLC) tumors extracted from patient studies. The tumors were segmented from the images and scaled in order to make the printing possible. PET images of the 3D printed inserts are displayed in Fig. 1. To achieve heterogeneous uptake, the inserts consist of two separate compartments that can be filled with different activity solutions. All three inserts reflect a unique uptake pattern, including homogeneous uptake (tumor 1), heterogeneity uptake across two compartments (tumor 2), and a tumor containing a necrotic core (tumor 3). Tumor 1 yields a size of 40.3 mm × 44 mm × 54.5 mm (volume 46.05 ml), the upper and lower part of tumor 2 yield sizes of 33.9 mm × 37 mm × 30 mm (volume 10.75 ml), and 24.3 mm × 40.5 mm × 36.6 mm (volume 13.12 ml), respectively. While the outer part of tumor 3 and the necrotic core yield sizes of 56 mm × 54 mm × 65.1 mm (volume 65.35 ml) and 25 mm × 24 mm × 31 mm (volume 7.8 ml). Tumor 1, the lower part of tumor 2, and the outer part of tumor 3 were filled with an activity solution of 19.49 kBq/ml, the upper part of tumor 2 with 10.94 kBq/ml, and the large background compartment of the NEMA IQ phantom with 1.94 kBq/ml. The necrotic core of tumor 3 contained nonradioactive water. The phantom was also scanned on a Siemens Biograph mCT40. Images were reconstructed using the same parameters as the IQ phantom described above (see also Table 1).

**Table 1:** List of used reconstruction parameters

Parameters	Variables
SBR	10:1, 5:1, 0.5:1, 0.25:1
Reconstruction	OSEM, TOF, PSF, PSFTOF
Matrix/voxel size	256 × 256 × 111 (3.1819 × 3.1819 × 2 mm), 400 × 400 × 111 (2 × 2 × 2 mm)
Scan duration	30 s, 60 s, 120 s, 300 s
FWHM	0, 2, 4, 6, 8 mm

### 2.B. Segmentation

Spheres and 3D printed inserts were segmented using low‐dose CT‐ and PET‐based delineation methods. The CT‐based volume of interest (VOI) of the spheres was generated by the manual placement of a sphere‐shaped VOI with corresponding sphere diameter, while the 3D printed inserts were manually segmented using an in‐house software developed for the analysis of PET images. The PET‐based segmentations were generated with a region growing method using a connectivity of 26 voxels implemented in Matlab 2014b (Mathworks, Natick, MA, USA). For the high uptake spheres and the tumor‐shaped inserts, the segmented region grew from the center voxel of the highest SUV_peak_ seed point till voxel intensities became less than 41% of this SUV_peak._ 20 Conversely, for the low uptake spheres the segmentation algorithm was inverted: the segmented region grew from the center voxel of the lowest SUV_peak_ seed point till the voxel intensities became larger than a SUV of 0.59. To prevent excessive overestimation of the actual sphere volume, the PET‐based segmentation was limited to a sphere volume of 300% of the CT‐segmented sphere volume. As texture analysis in three dimensions requires the VOI to be specified in all three spatial dimensions, only those segmentations that eventually resulted in an actual 3D VOI were considered for feature extraction (i.e., segmentations of 1 or 2 voxels or those located in a single image plane were discarded).

### 2.C. Radiomic feature extraction

Image processing and feature extraction were performed using Matlab 2014b. For each VOI, 246 radiomic features were calculated, including 19 morphological features, 3 local intensity features, 18 statistical features, and 206 textural features (100 gray level co‐occurrence‐based features, 64 gray level run length‐based features, 32 gray level size zone‐based features, and 10 neighborhood gray tone difference‐based features).(18) All calculated features are listed in the supplemental materials (Table S1). Textural features were extracted from discretized image stacks that reduced the continuous‐scaled SUV to a countable number of intensity values. Image stacks were discretized using a fixed number of 64 bins (FBN) and a fixed bin width (FBW) of 0.25 for the high uptake spheres and the 3D prints. For the low uptake spheres (SBR <1), a bin width of 0.05 was applied. Images were analyzed in both two and three dimensions with a connectivity of 8 and 26 voxels, respectively (using a Chebyshev norm of 1). Single feature values derived from the gray level co‐occurrence and gray level run length matrices were calculated by both averaging the obtained feature values over all directions and by extracting the features directly from a single merged matrix in which the gray level co‐occurrence or gray level run length matrices over all directions were summed. We ensured that image processing and feature calculation matched publicly available benchmark values of digital phantom and patient test data.^21^

### 2.D. Feature clustering

The number of radiomic features is usually high in comparison to the number of subjects included in a PET study. In order to avoid overfitting, the feature space has to be reduced before features can be used for classification or other purposes. In this study, clusters of features with the same properties were identified using a Spearman correlation matrix of the CT‐segmented features, evaluating the monotonic relationship between features. The correlation matrix was ordered by minimizing the mean correlation difference between neighboring features. A cluster was defined by features that resulted in a high correlation, that is, that had mutual Spearman's correlation coefficients of >0.7.^22^

We have determined whether the composition of feature clusters was affected by discretization, reconstruction algorithm, sphere size, and activity uptake. For defining the correlation matrices, we used the default settings: all activity uptakes and sphere sizes, a European Association of Nuclear Medicine Research Ltd (EARL) compliant reconstruction (OSEM, 4 mm FWHM, 120 s scan duration),^23^ matrix size 256 × 256 × 111, CT‐based segmentation, and FBW discretization. The clusters of this default correlation matrix were compared with the clusters of other correlation matrices which were composed on the basis of different settings for discretization (FBW and FBN) and reconstruction (OSEM and PSF). Subsequently, the data of the default setting were divided into four subcategories: larger (diameters of 37, 28 and 22 mm) — high uptake spheres (SBR >1), larger — low uptake spheres (SBR <1), smaller (diameters of 17, 13 and 10 mm) — high uptake spheres, and smaller — low uptake spheres. In this case, all clusters were compared against the clusters of the default correlation matrix of the larger high uptake spheres. Moreover, we have compared the clusters of all statistically equal replicates using the default settings to ensure that all found differences in the composition of feature clusters could be ascribed to the sources of variation.

### 2.E. Repeatability analysis

Repeatability was evaluated using the intraclass correlation coefficient (ICC), calculated with the irr package (version 0.84), available from the Comprehensive R Archive Network (http://www.r-project.org). A two‐way single measure model was used to evaluate the consistency of the replicates of each setting. The ICC is the ratio of the intercluster variance and the total variance, that is, the sum of the intracluster and intercluster variability. Intraclass correlation coefficient values lie between 0 and 1 representing perfect repeatability. Furthermore, a high ICC indicates a high intercluster variance in comparison with the intracluster variance. Therefore, features yielding a high ICC are also sensitive to insert‐specific differences.

Before extracting the ICCs, the data were split into the same four different underlying data subcategories that were used for the redundancy analysis. The 3D inserts are forming an additional subcategory. The ICC was calculated for every combination of subcategory, matrix size, reconstruction algorithm, scan duration, Gaussian filter, discretization method, and segmentation method. Each sphere with a different size or SBR, as well as each 3D insert, was considered a different subject. The equivalent replicates were regarded as the different raters. Features exhibiting an ICC >0.8 were considered to represent good repeatability.^24^ For each setting, the percentage of repeatable features was obtained to identify trends in the data. Smaller subsets of features were analyzed in order to avoid that large groups of features with similar properties overrepresented and biased the analysis. For this purpose, we used a predefined set of uncorrelated radiomic features, as identified previously (Table 2, Supplemental Figures [Link], [Link], [Link], [Link]).^9^

**Table 2:** Predefined feature set used for repeatability analysis shown in Supplemental Figures [Link], [Link], [Link], [Link]

Selection of uncorrelated features
Homogeneity_3D_ (NGLDM)
Energy
Short runs emphasis_3D_
Gray level nonuniformity_3D_
Skewness
Long zone low gray level emphasis_3D_
Long run emphasis_3D_
Low gray level run emphasis_3D_
High gray level run emphasis_3D_
SUVmax
Busyness

To investigate the potential relationship between the repeatability of radiomic features and image noise, a variance image of the statistically equal replicates was calculated for every studied setting. The image noise was measured by calculating the coefficient of variation over four different spherical VOIs defined in the phantom background of the variance image.

## 3 Results

### 3.A. Feature clustering

**Figure 2:**
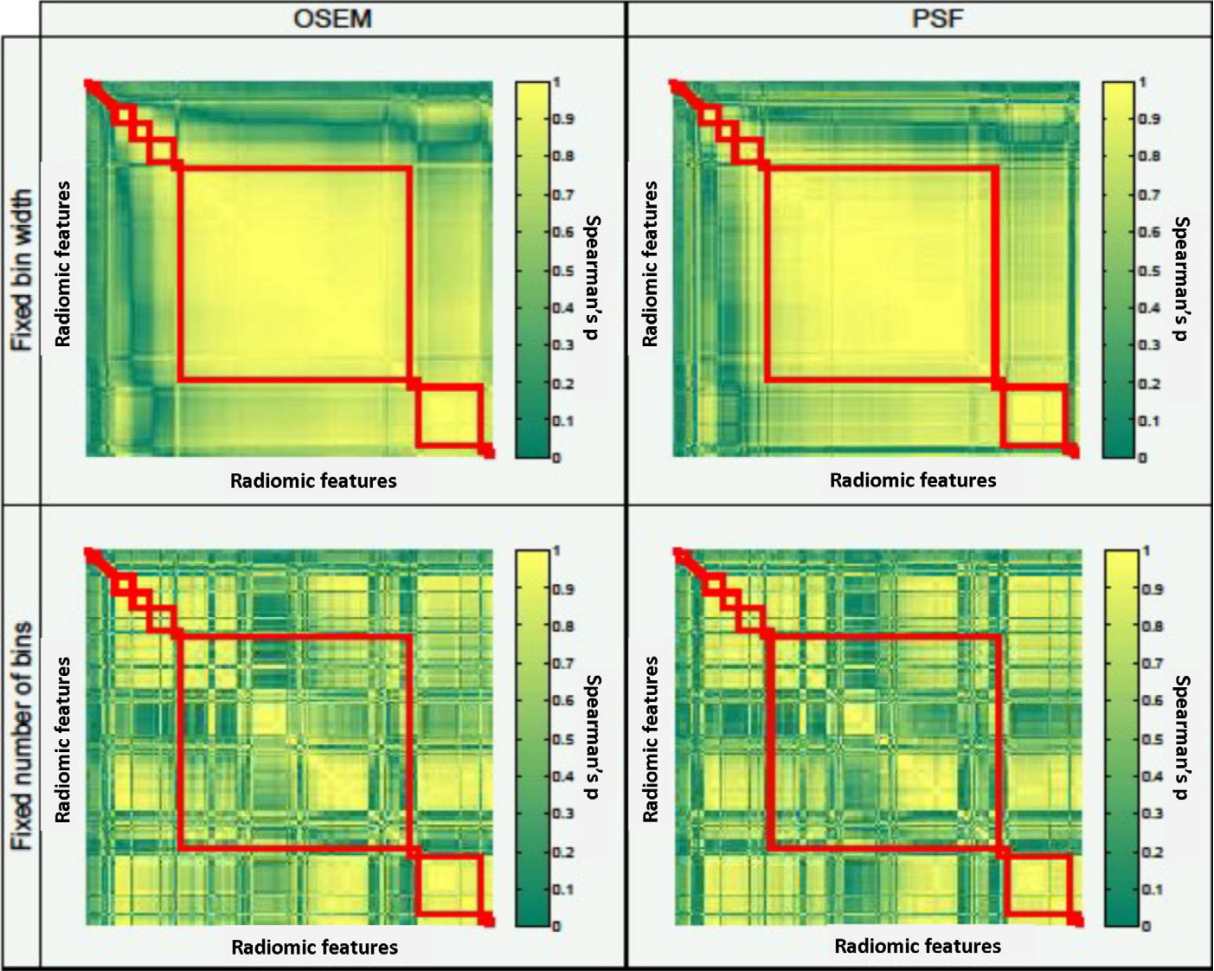
Impact of discretization and reconstruction setting on the composition of feature clusters: feature clusters (red rectangles) were defined based on Spearman's correlation matrices. The default setting in the upper left corner consists of all activity uptakes and sphere sizes, matrix size 256 × 256 × 111, OSEM reconstruction, 120 s scan duration, FBW discretization, 4 mm FWHM, and CT‐based segmentation. The feature order of this setting was also used to display the correlation matrices of the other settings. [Color figure can be viewed at wileyonlinelibrary.com]

**Figure 3:**
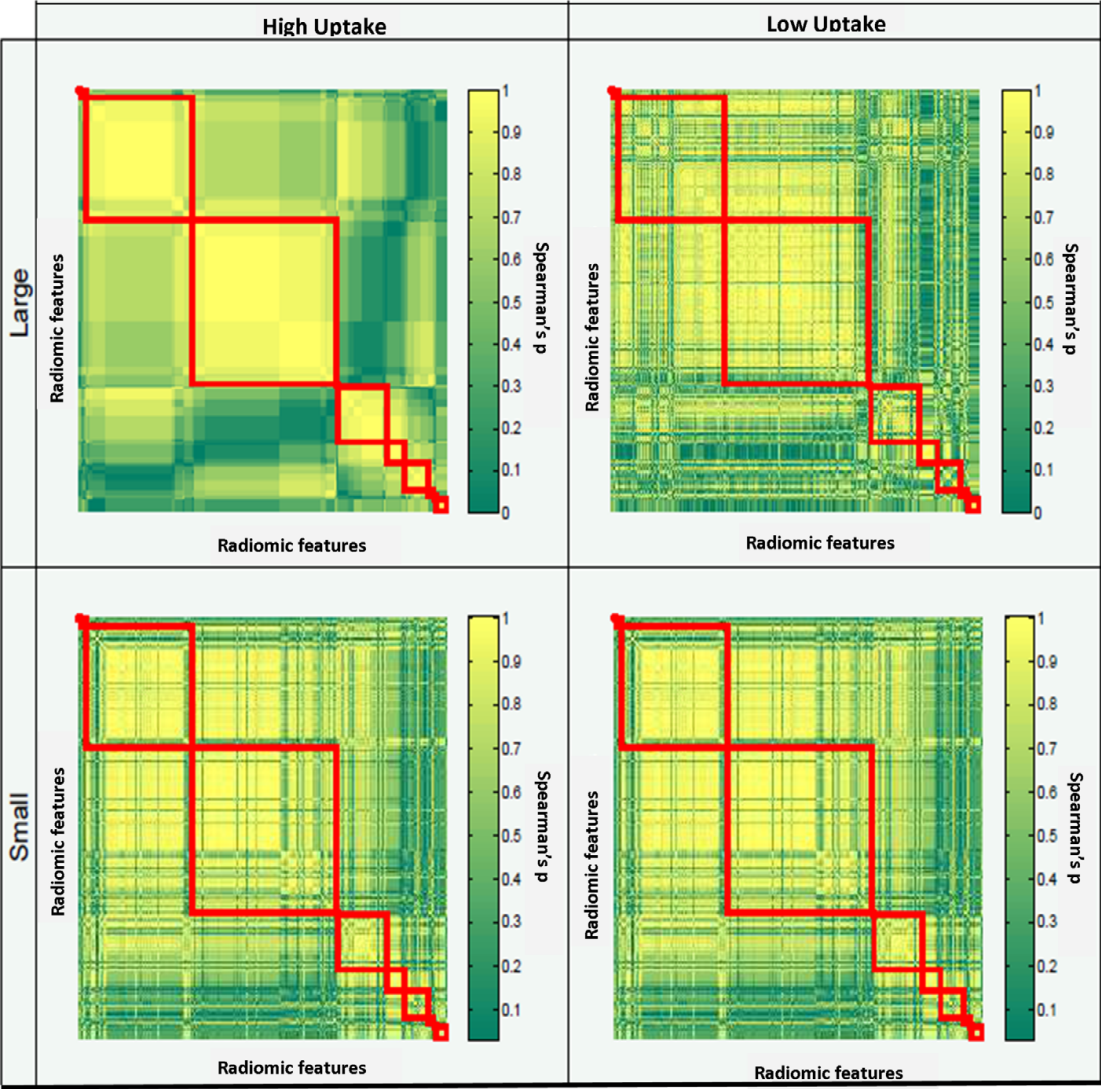
Impact of sphere size and activity uptake on the composition of feature clusters: feature clusters (red rectangles) were defined based on Spearman's correlation matrices. The default setting in the upper left corner consists of the data of larger high uptake spheres, EARL reconstruction, matrix size 256 × 256 × 111, 120 s scan duration, FBW discretization, and CT‐based segmentation. The feature order of this setting was also used to display the correlation matrices of the other settings. [Color figure can be viewed at wileyonlinelibrary.com]

Figures 2 and 3 demonstrate how the Spearman's correlation matrix was affected by reconstruction algorithm and discretization method (Fig. 2), as well as by sphere size and activity uptake (Fig. 3). In order to illustrate the differences in correlation, the feature order and cluster composition of the default setting were used to display the correlation matrices of the other settings. The correlation matrix of this setting is displayed in the upper left corner of each figure. Changing the reconstruction algorithm to PSF had a minor impact on the correlation matrix. However, the increased number of clusters being composed of features with mutual Spearman's correlation coefficients of <0.7 demonstrates that the impact of the discretization method was much larger. Similarly, Fig. 2 shows that sphere size and activity uptake both had a major impact on the correlation matrix. The correlation matrices of the statistically equal replicates showed to be similar, and therefore all found differences in the composition of feature clusters could be ascribed to the sources of variation.

### 3.B. Repeatability analysis

**Figure 4:**
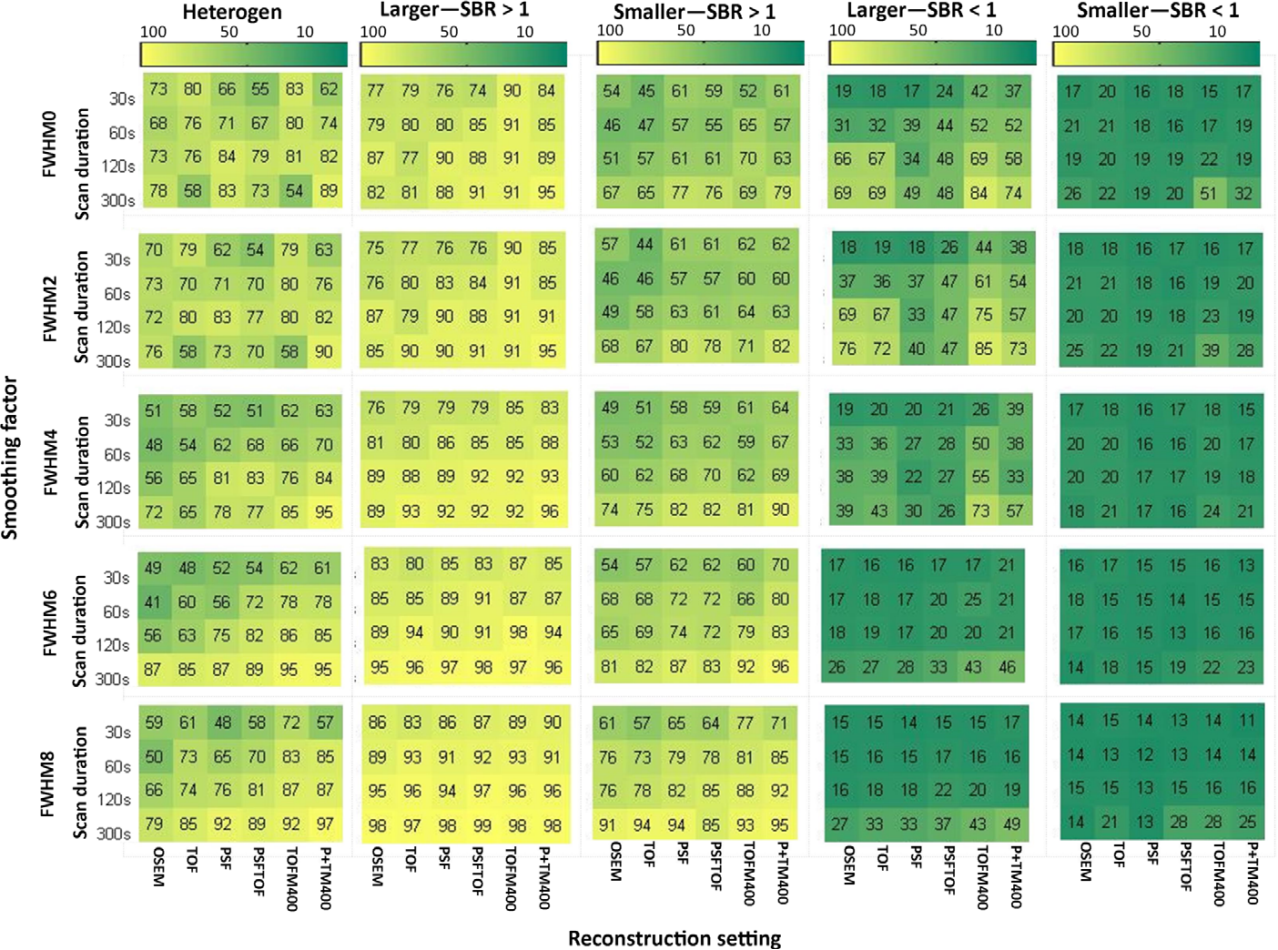
Percentage of repeatable features discretized with FBW: Percentage of all features discretized with FBW and segmented based on CT exhibiting an ICC >0.8 for all studied settings and underlying data categories (from left to right: heterogeneous 3D prints, bigger spheres with high uptake, smaller spheres with high uptake, bigger spheres with low uptake, and smaller spheres with low uptake). TOFM400/P+TM400: TOF/PSF+TOF reconstruction with matrix size 400 × 400. [Color figure can be viewed at wileyonlinelibrary.com]

**Figure 5:**
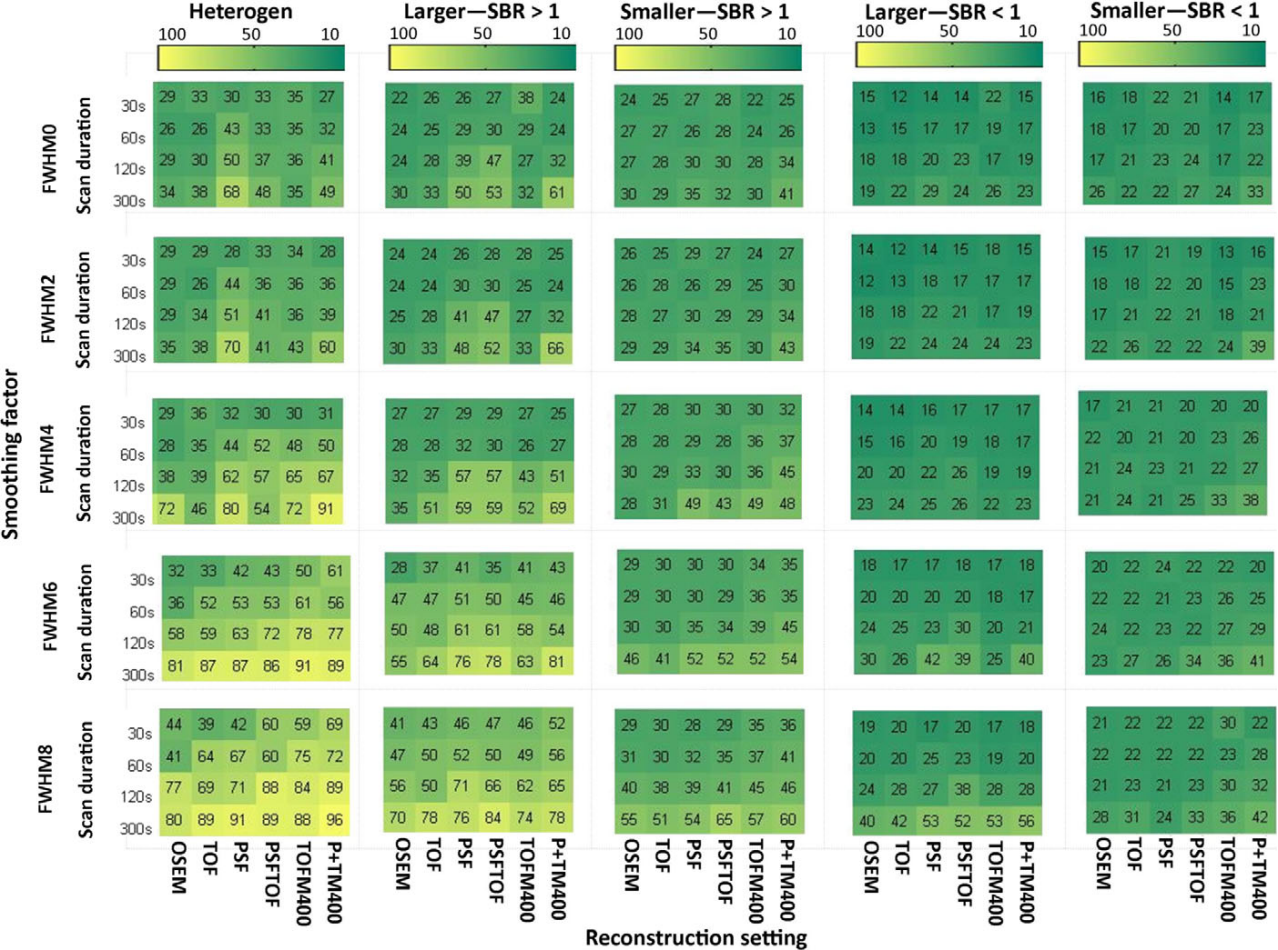
Percentage of all features discretized with FBN: Percentage of all features discretized with FBN and segmented based on CT exhibiting an ICC >0.8 for all studied settings and underlying data categories (from left to right: heterogeneous 3D prints, bigger spheres with high uptake, smaller spheres with high uptake, bigger spheres with low uptake, and smaller spheres with low uptake). TOFM400/P+TM400: TOF/PSF+TOF reconstruction with matrix size 400 × 400. [Color figure can be viewed at wileyonlinelibrary.com]

**Figure 6:**
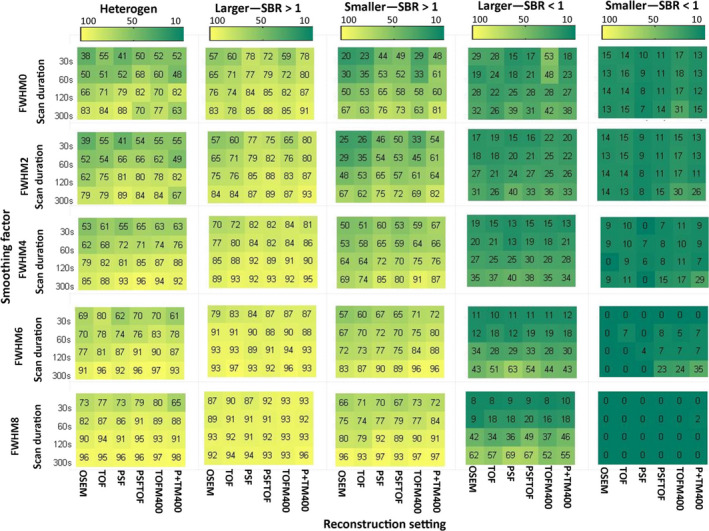
Percentage of repeatable features discretized with FBW: Percentage of all features discretized with FBW and segmented based on PET exhibiting an ICC >0.8 for all studied settings and underlying data categories (from left to right: heterogeneous 3D prints, bigger spheres with high uptake, smaller spheres with high uptake, bigger spheres with low uptake, and smaller spheres with low uptake). TOFM400/P+TM400: TOF/PSF+TOF reconstruction with matrix size 400 × 400. [Color figure can be viewed at wileyonlinelibrary.com]

**Figure 7:**
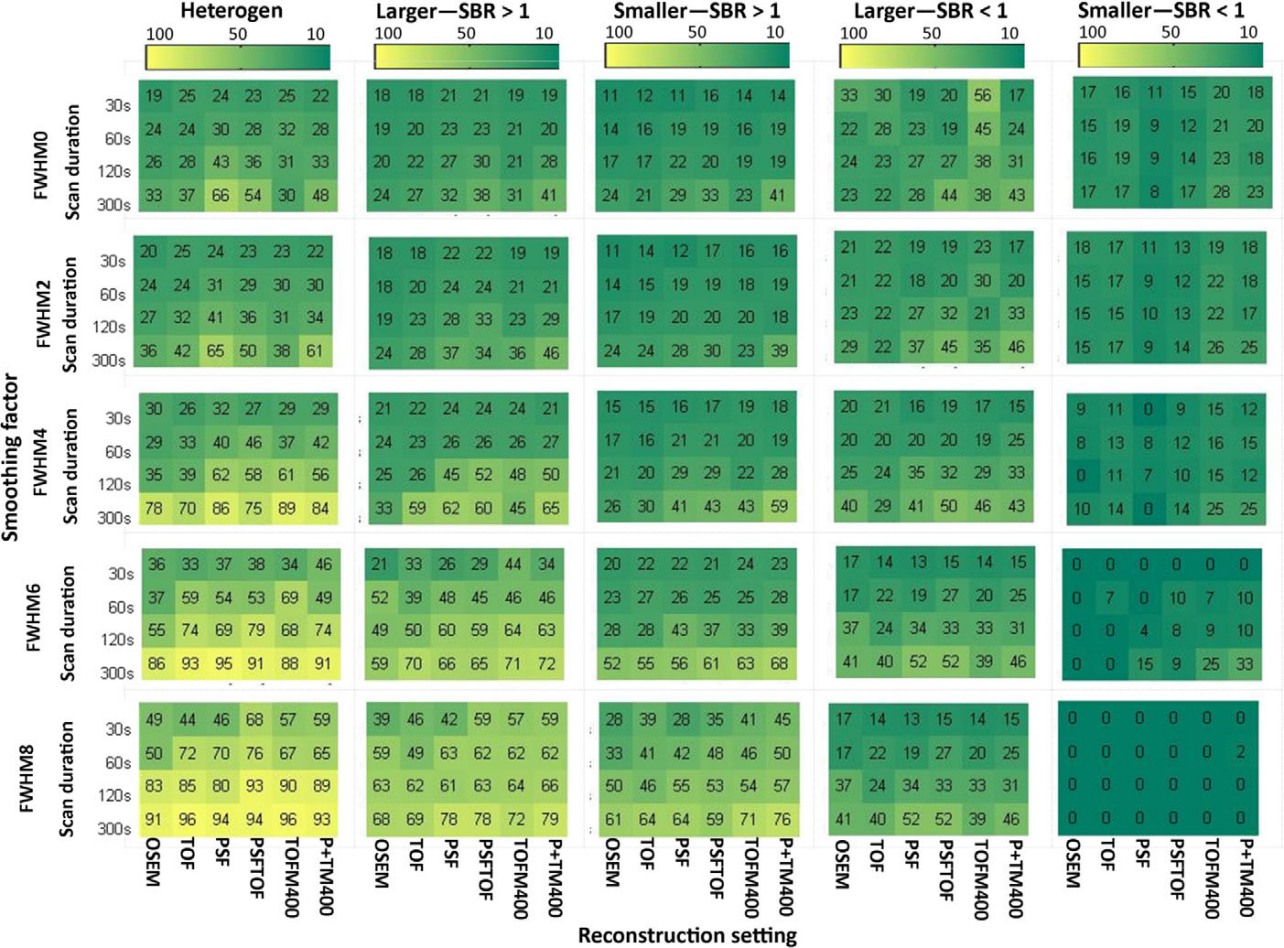
Percentage of repeatable features discretized with FBN: Percentage of all features discretized with FBW and segmented based on PET exhibiting an ICC >0.8 for all studied settings and underlying data categories (from left to right: heterogeneous 3D prints, bigger spheres with high uptake, smaller spheres with high uptake, bigger spheres with low uptake, and smaller spheres with low uptake). TOFM400/P+TM400: TOF/PSF+TOF reconstruction with matrix size 400 × 400. [Color figure can be viewed at wileyonlinelibrary.com]

Repeatability analysis was not performed for 15 geometry features derived from the CT‐based segmentation, as they are a function of sphere size and hence exhibit an ICC of 1 by definition. The ICC values of every calculated feature for discretization with FBW and FBN are listed in the supplemental material (Tables S1 and S2). Figures 4 and 5 display how the repeatability of radiomic features is affected by heterogeneity, activity uptake, sphere size, discretization method, image noise, reconstruction algorithm, and matrix/voxel size for CT‐based segmentations. The impact of the same sources of variation for PET‐based segmentations is displayed in Figs. 6 and 7.

**Figure 8:**
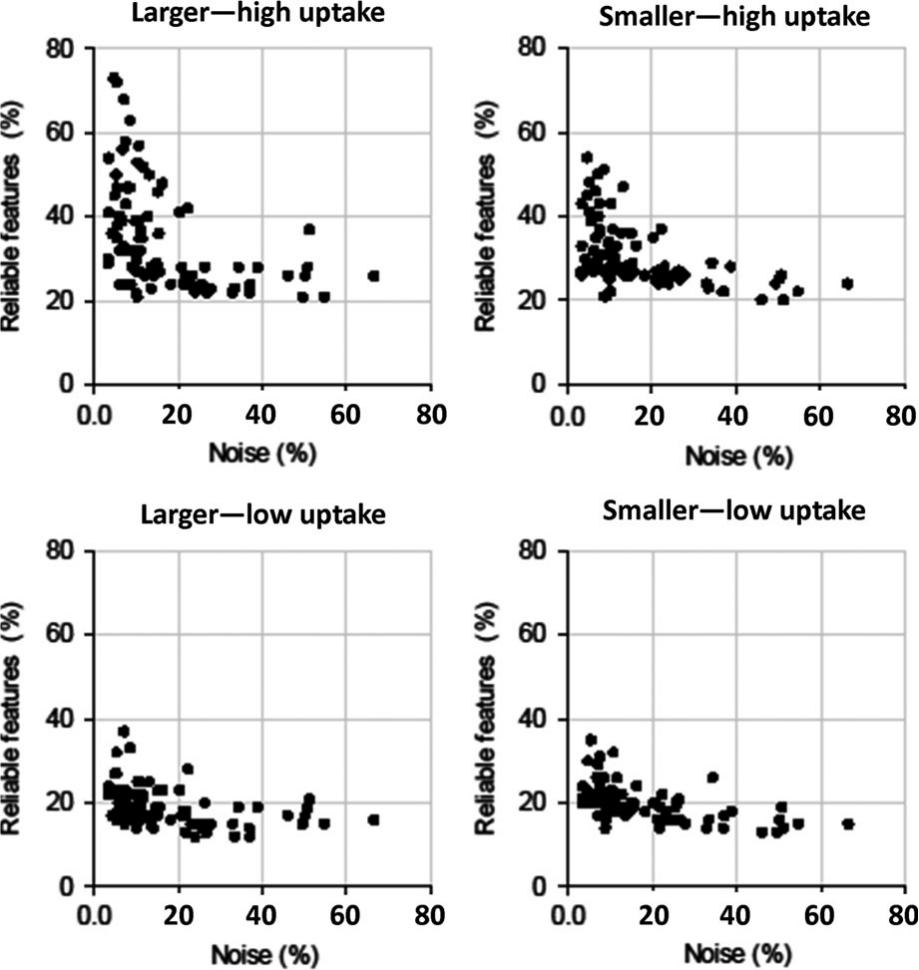
Influence of noise on number of repeatable features extracted from NEMA IQ phantom scan with SBR 1:10 for all reconstructions, scan durations, smoothing factors, and matrix sizes: Number of repeatable features in the different subcategories as function of image noise

In general, underlying data, image noise, and discretization method had a high impact on feature repeatability. The reconstruction setting had also a big influence, when FBN discretization was applied. Regarding the underlying data, bigger spheres yielded more repeatable features than smaller spheres and spheres with high activity uptake yielded more repeatable features than spheres with low activity uptake. In terms of repeatability, 3D printed inserts showed comparable results with those of larger high uptake spheres. Image noise reduction in terms of longer scan durations and applying smoothing to the images resulted in better repeatability, although the effects of smoothing depended on the segmentation and discretization method. The inverse proportional relationship between number of repeatable features and noise is illustrated in Fig. 8. This figure shows the number of repeatable features discretized with FBN for the NEMA IQ phantom scan with SBR 1:10 for all reconstruction methods, scan durations, smoothing factors, and matrix sizes as function of noise. The impact of image noise depended on the used discretization method: For FBN discretization (Figs. 5 and 7), the number of repeatable features increased with applied smoothing. For FBW discretization of high uptake spheres and 3D prints, the effect of smoothing was marginal (Figs. 4 and 6), while for low uptake spheres, an increase in smoothing even led to less repeatable features. In particular when FBN and PET‐based segmentations are used in combination, mitigation of noise by smoothing seems to be beneficial in terms of more repeatable features, while for FBW and CT‐based segmentation smoothing as means of noise reduction seems less effective. Both discretization methods yielded in general different repeatability pattern: FBW discretization led to better repeatability and to less variation across reconstruction algorithms. While for FBN discretization, differences across reconstruction algorithm were mainly observed for longer (120 and 300 s) scan durations. For those scan durations, the repeatability was the lowest for images reconstructed with OSEM or TOF and increased by adding PSF.

On the other hand, the used matrix size as well as the segmentation method had only minor impact on repeatability: Changes in matrix size led mainly to differences for the heterogeneous inserts and the low uptake spheres, where a bigger matrix size (i.e., a smaller voxel size) resulted in more repeatable features. Also differences between segmentation methods were mainly observed for the low uptake data. For the smaller low uptake spheres and PET‐based segmentations, the number of repeatable features was lower than for CT‐based segmentations and decreased even more with increasing smoothing. An overview of the parameters leading to the best repeatability behavior for the different activity uptake groups is listed in Table 3.

**Table 3:** Parameters/methods/settings leading to best repeatability for different activity uptake groups

	Heterogeneous inserts	High uptake spheres	Low uptake spheres
Reconstruction	Minor impact	Minor impact	PSF+TOF
Matrix/voxel size	Minor impact	Minor impact	Smaller voxel size (2 × 2 × 2 mm)
Scan duration	300 s	300 s	300 s
Smoothing (FWHM)	6/8 mm	6/8 mm	0 mm
Discretization method	FBW (bin width 0.25)	FBW (bin width 0.25)	FBW (bin width 0.05)
Segmentation method	Minor impact	Minor impact	CT‐based segmentation

### 3.C. Repeatable features

Repeatable features for all subcategories and the EARL‐compliant reconstruction are listed in Table 4 for both discretization methods. Even though every subcategory resulted in more repeatable features for FBW discretization, the number of features found to be repeatable for all subcategories is comparable for both discretization methods with a big overlap: Five features were found to be repeatable only for FBW discretization, two only for FBN discretization, sixteen features for both methods, and two features do not require the discretization step.

**Table 4:** Features with an ICC >0.8 for all subcategories and EARL‐compliant reconstruction for both discretization methods

FBN	FBW
Integrated intensity (does not require discr.)	Integrated intensity (does not require discr.)
Energy intensity (does not require discr.)	Energy intensity (does not require discr.)
Joint‐maximum_2D_avg_	Joint‐maximum_2D_avg_
Joint‐entropy_2D_avg_	Joint‐entropy_2D_avg_
Difference‐entropy_2D_avg_	Difference‐entropy_2D_avg_
Sum‐entropy_2D_avg_	Sum‐entropy_2D_avg_
Angular‐second‐moment_2D_avg_	Angular‐second‐moment_2D_avg_
Joint‐entropy_2D_comb_	Joint‐entropy_2D_comb_
First measure of information correlation_3D_avg_	
	Gray level nonuniformity GLRLM_2D_avg_
Run length nonuniformity_2D_avg_	Run length nonuniformity_2D_avg_
Run‐entropy_2D_avg_	Run‐entropy_2D_avg_
	Gray level nonuniformity GLRLM_2D_comb_
Run length nonuniformity_2D_comb_	Run length nonuniformity_2D_comb_
Run entropy_2D_comb_	Run entropy_2D_comb_
Gray level nonuniformity GLRLM_3D_avg_	Gray level nonuniformity GLRLM_3D_avg_
Run length nonuniformity_3D_avg_	Run length nonuniformity_3D_avg_
Gray level nonuniformity GLRLM_3D_comb_	Gray level nonuniformity GLRLM_3D_comb_
Run length nonuniformity_3D_comb_	Run length nonuniformity_3D_comb_
	Gray level nonuniformity normalized GLSZM_2D_
Zone size nonuniformity_2D_	Zone size nonuniformity_2D_
Zone size entropy_2D_	Zone size entropy_2D_
Zone size nonuniformity_3D_	
	Gray level nonuniformity GLSZM_3D_
	Coarseness_3D_

## 4 Discussion

This study demonstrated that both dimensionality reduction and repeatability of ^18^F‐FDG PET radiomic features are sensitive to most sources of variation. In the subsequent sections, the underlying trends are described in more detail.

### 4.A. Feature clustering

As described in several other studies,^9^, ^25^ we found that many features were highly correlated. Discretization, sphere size, and activity uptake had a major impact on this correlation, while reconstruction method had less influence. To reduce the feature space, representative features should be chosen from each cluster. We showed that the composition of the correlation matrices was repeatable, but dependent on various factors such as image discretization, activity uptake, and sphere size. As a consequence, these correlation matrices yield different clusters of correlated features. Therefore, the representative features extracted from these clusters will differ across these matrices. Hence, the outcome of redundancy analyses is only generalizable among studies when these studies applied similar settings.

### 4.B. Feature repeatability

In this study, radiomic features extracted from larger high uptake spheres (SBR >1) generally showed higher repeatability than those extracted from smaller low uptake spheres (SBR <1). In a clinical setting, the tracer uptake activity is affected by tumor type and uptake mechanism. Furthermore, the signal depends on the used PET isotope. This can result in images with a poor signal‐to‐noise ratio (e.g., ^89^Zr‐antibodies in immunoPET studies) or even in very low uptake areas (lower than surrounding background). Therefore, it is not recommended to generalize results of radiomic studies in different tumor types and PET tracers, as most studies so far explored the performance of radiomic features on FDG PET/CT studies.

In radiomic studies, both discretization methods (FBW as well as FBN) are widely used. However, several studies suggest the use of FBW discretization as better clinical applicability and repeatability has been shown.^21^, ^26^, ^27^ Furthermore, Orlhac et al. demonstrated that features discretized with FBW led to more significant differences in feature values across tumor types and hence to more meaningful results.^19^ Our findings also support the use of FBW discretization, as it led in general to a larger number of repeatable features (yielding a high ICC) for both phantoms and hence also to more features sensitive to heterogeneity information.

Previous studies reported high variability of feature values across reconstruction algorithm^11^, ^14^ for images discretized with FBN. Our results confirm that FBN discretization led also to higher variation in repeatability performance across reconstruction algorithm. A reason for this effect might be that for this setting, the bin width is sensitive to image noise and therefore every image is discretized with a different bin width. This hypothesis is in line with the finding that decreasing image noise by image smoothing resulted in increased number of repeatable features mostly for FBN discretization. Another point that supports this hypothesis is that also the combination of FBN discretization and PET‐based segmentation resulted in an increase in number of repeatable features, when compared with CT‐segmentation. This is likely due to the fact that the 41% SUV_peak_ method eliminates outliers from the region of interest. Therefore, the intensity ranges across regions of interests (and also the bin width) become comparable across images and lead therefore to an increase in repeatability.

Our results suggest that a large number of features are sensitive to image noise. In the majority of the cases, increased smoothing resulted in a higher number of repeatable features (Figs. 4 and 5), and these effects are most pronounced when using PET‐based segmentation in combination with FBN discretization (Fig. 7). This may seem counter‐intuitive as for smoothing there is a trade‐off between noise and spatial resolution; that is, increased smoothing leads to less noise but lower spatial resolution and possibly less observable uptake heterogeneity. PET textural features, capturing intensity differences between neighboring voxels, can be highly sensitive to stochastic image variation.^12^ As the reduction of noise leads to more homogeneous image texture, this may lead to more comparable textural matrices across the statistically equal replicates and hence to higher repeatability. A drawback of decreasing image noise by image smoothing might be that important textural information describing tumor uptake heterogeneity might get lost. In our study, however, for high uptake data and 3D printed heterogeneous inserts, actually more features showed good repeatability with increasing smoothing and/or for longer scan duration. This indicates that for the heterogeneous phantom insert data, increasing smoothing did not necessarily eliminate important heterogeneity information. Therefore, noise mitigation by increasing scan statistics and/or by image smoothing could be a valid option and should be further explored. As the 3D printed inserts contain only coarse heterogeneity information, these findings can only be applied to tumors showing similar heterogeneity pattern as the 3D printed inserts. For tumors showing subtle heterogeneity uptake, smoothing might affect the heterogeneity information and therefore also influence the repeatability behavior of radiomic features. Furthermore, in this study, it was impossible to assess the impact of smoothing on the sensitivity of feature values to underlying biological factors.

On the other hand, low uptake spheres (SBR <1) discretized with FBW resulted in lower repeatability for higher levels of smoothing. In this case, smoothing decreased the intensity range in the spheres and therefore the chosen bin width can become inappropriate. Therefore, for low activity uptake, the bin width should be chosen carefully and/or the use of smoothing should be applied with care and/or avoided all together.

Several studies showed that a large number of features exhibit high variability across various reconstruction settings.^13^, ^15^, ^28^ Our study showed that especially for FBN discretization the number of repeatable features also depended on the used reconstruction algorithm. For example, images reconstructed with PSF or PSF + TOF yielded higher repeatability than OSEM or TOF reconstructions. The higher repeatability found by using PSF is consistent with the fact that PSF decreases image noise^29^ and with the previously reported finding that image noise and repeatability have an inverse proportional relationship. Moreover, the additional use of TOF improves image quality and reduces image noise^30^ and is therefore expected to increase feature repeatability. However, our results showed the same repeatability for images reconstructed with OSEM and images reconstructed with TOF. A comparison of image noise between these images showed that the TOF‐effect on our scanner and for this phantom was small and had therefore also only a small effect on the repeatability of features.

Many studies reported on differences in feature values across different voxel sizes.^31^, ^32^ In our study, differences in repeatability were mainly observed for the tumor‐like inserts. Here, a smaller voxel size resulted in more repeatable features. A possible explanation might be that the smaller voxel size can capture heterogeneity information more precisely and it is therefore preferable to follow the recent recommendation by Hatt et al. to apply a standardized voxel size of 2 × 2 × 2 mm^8^. The latter is also recommended because the value of some features depends on the number of voxels within a given VOI.

Previous studies reported a high variability of feature values across different segmentation results.^27^, ^28^, ^33^, ^34^ However, as demonstrated by Hatt et al.^33^ even though different segmentations lead to a variability in feature values, their predictive value might not change. Our results indicate that the 41% SUV_peak_ segmentation algorithm leads to good repeatability for a large number of features in line with,^34^ although it does not lead to reliable segmentations in all cases.^35^ In our study, differences in repeatability were mainly observed in the low uptake data. As explained before, for PET‐segmentations, image noise influences not only the repeatability of radiomic features but also the quality of the segmentation. Therefore, the lower number of repeatable features in the low uptake data might be caused by poor segmentation results due to image noise.

### 4.C. Repeatable features

Only a small number of features were identified to be repeatable for all subcategories. The majority of these features were repeatable for both discretization methods. Some of these features (gray level nonuniformity run length, run length nonuniformity) were identified before to be insensitive to the discretization step.^9^ The high ICC also indicates that these features are informative regarding differences in tracer uptake heterogeneity. This is in line with previous studies showing that, for example, coarseness contains valuable information about survival for NSCLC patients or response to therapy for esophageal cancer patients.^36^, ^37^ While sum and difference entropy (GLCM) showed to have prognostic value for NSCLC tumors.^38^, ^39^, ^40^

A drawback of our study is that only phantom data were included, although we attempted to make the study as clinically relevant as possible by using 3D printed phantom inserts reflecting heterogeneous tumor uptake pattern. It should be noted that our study was designed to explore the technical performance of radiomic features under controlled experimental conditions, thereby avoiding biological uncertainties or variations in imaging procedures. Yet, it is of interest to perform, for example, repeatability studies which include these factors to further test radiomic performance under clinical conditions. Our results show that it may be warranted to collect these repeatability studies for various diseases and tracers as the tracer bio distribution and tumor uptake can be very different among patient groups and tracers. We showed that differences in size, level, and intratumoral distribution of tracer uptake have a large effect on radiomic feature repeatability and thus on the optimal settings to be used in a radiomics analysis pipeline.

## 5 Conclusion

This study reports on the impact of underlying data, image reconstruction methods and settings, noise, discretization method, and delineation method on the dimensionality reduction and repeatability of ^18^F‐FDG PET radiomic features, which is an important measurement of error. Our data show that feature reduction is sensitive to discretization, sphere size, and activity uptake, and is therefore only generalizable among studies using the same settings. This study demonstrates that clinical PET studies and examinations need to be standardized in order to use ^18^F‐FDG PET radiomics as quantitative imaging biomarkers. Although this conclusion is not new for standard quantitative PET biomarkers, our study suggests that, in particular for radiomics features, efforts should focus on noise reduction sometimes even at the cost of spatial resolution and optimizing the choice for image reconstruction method, discretization method, and segmentation method. For every clinical application, radiotracer and disease type, a validation of radiomic feature performance/repeatability needs to be performed as its performance depends on the nature of the underlying data, that is, as function of tumor size, shape, tracer uptake level, contrast, and intratumoral uptake distribution.

## Conflicts of interest

The authors have no relevant conflicts of interest to disclose.

## Supporting information


**Fig. S1.** Percentage of repeatable features discretized with FBW: Percentage of representative features discretized with FBW and segmented based on CT exhibiting an ICC > 0.8 for all studied settings and underlying data categories (from left to right: heterogeneous 3D prints, bigger spheres with high uptake, smaller spheres with high uptake, bigger spheres with low uptake, and smaller spheres with low uptake). TOFM400/P+TM400: TOF/PSF+TOF reconstruction with matrix size 400 × 400.

**Fig. S2.** Percentage of repeatable features discretized with FBN: Percentage of representative features discretized with FBN and segmented based on CT exhibiting an ICC > 0.8 for all studied settings and underlying data categories (from left to right: heterogeneous 3D prints, bigger spheres with high uptake, smaller spheres with high uptake, bigger spheres with low uptake, and smaller spheres with low uptake). TOFM400/P+TM400: TOF/PSF+TOF reconstruction with matrix size 400 × 400.

**Fig. S3.** Percentage of repeatable features discretized with FBW: Percentage of representative features discretized with FBW and segmented based on PET exhibiting an ICC > 0.8 for all studied settings and underlying data categories (from left to right: heterogeneous 3D prints, bigger spheres with high uptake, smaller spheres with high uptake, bigger spheres with low uptake, and smaller spheres with low uptake). TOFM400/P+TM400: TOF/PSF+TOF reconstruction with matrix size 400 × 400.

**Fig. S4.** Percentage of repeatable features discretized with FBN: Percentage of representative features discretized with FBN and segmented based on PET exhibiting an ICC > 0.8 for all studied settings and underlying data categories (from left to right: heterogeneous 3D prints, bigger spheres with high uptake, smaller spheres with high uptake, bigger spheres with low uptake, and smaller spheres with low uptake). TOFM400/P+TM400: TOF/PSF+TOF reconstruction with matrix size 400 × 400.

**Table S1.** ICC values for all features; extracted from EARL‐compliant reconstruction (OSEM, M256, 4 mm FWHM, CT‐based segmentation, FBW discretization).
**Table S2.** ICC values for all features; extracted from EARL‐compliant reconstruction (OSEM, M256, 4 mm FWHM, CT‐based segmentation, FBN discretization).
