# Supplementary figures and images for: Repeatability of 18F‐FDG PET radiomic features: A phantom study to explore sensitivity to image reconstruction settings, noise, and delineation method
Source: Med Phys. 2018 Dec 28;46(2):665–78. doi: 10.1002/mp.13322 (PMC7380016; doi:10.1002/mp.13322)

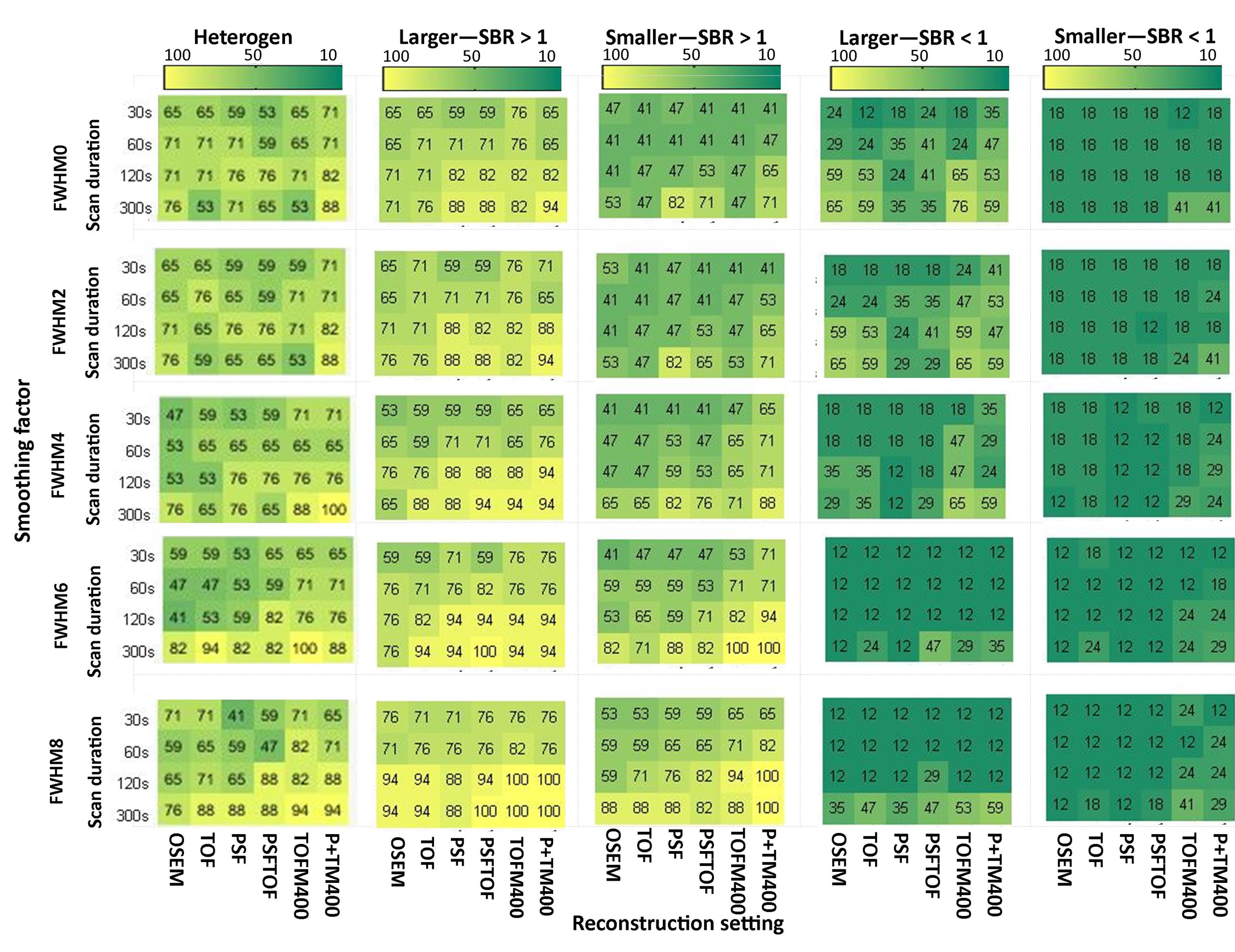

Supplement: Supplementary file 1 — Fig. S1. Percentage of repeatable features discretized with FBW: Percentage of representative features discretized with FBW and segmented based on CT exhibiting an ICC > 0.8 for all studied settings and underlying data categories (from left to right: heterogeneous 3D prints, bigger spheres with high uptake, smaller spheres with high uptake, bigger spheres with low uptake, and smaller spheres with low uptake). TOFM400/P+TM400: TOF/PSF+TOF reconstruction with matrix size 400 × 400. [file MP-46-665-s001.tif]

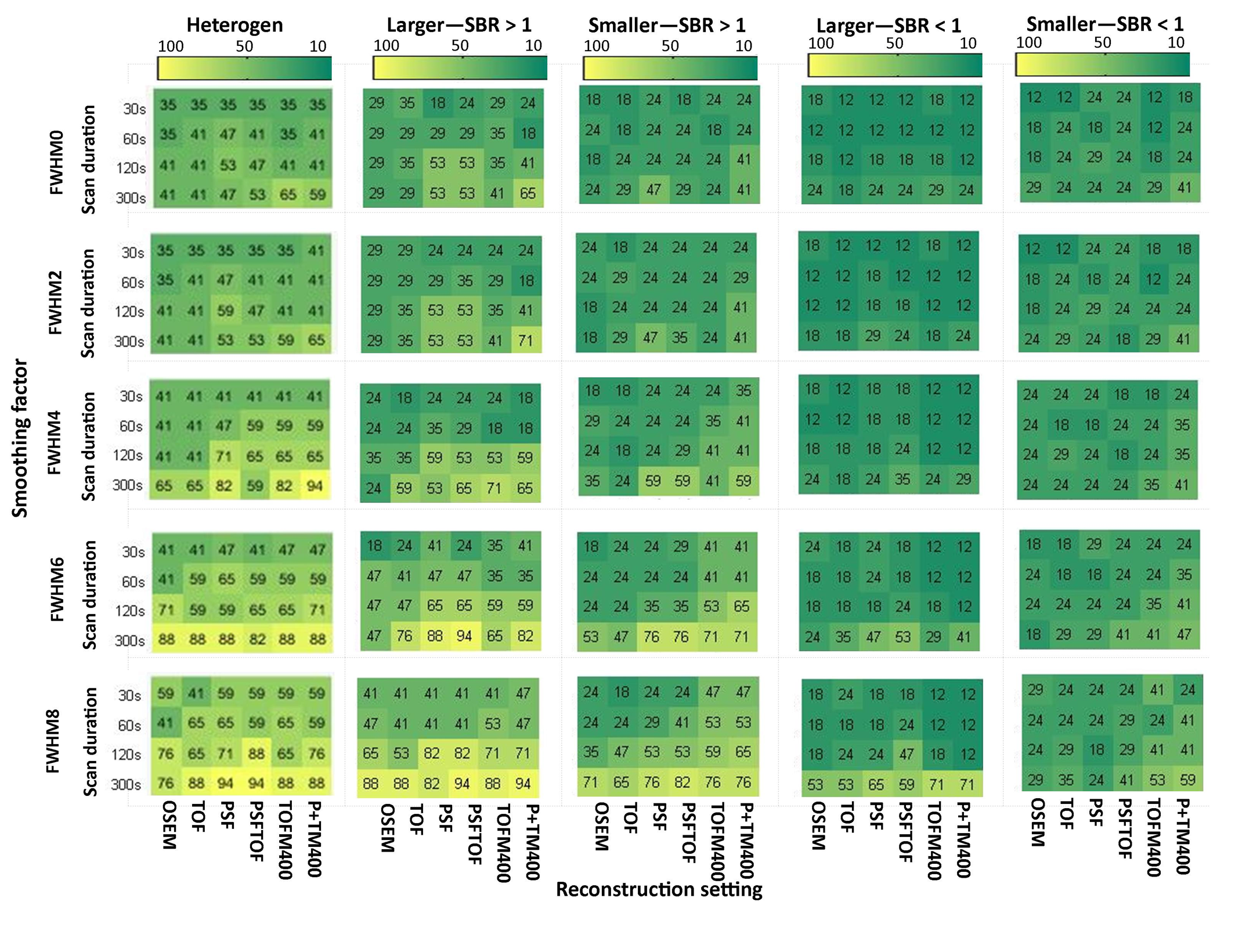

Supplement: Supplementary file 2 — Fig. S2. Percentage of repeatable features discretized with FBN: Percentage of representative features discretized with FBN and segmented based on CT exhibiting an ICC > 0.8 for all studied settings and underlying data categories (from left to right: heterogeneous 3D prints, bigger spheres with high uptake, smaller spheres with high uptake, bigger spheres with low uptake, and smaller spheres with low uptake). TOFM400/P+TM400: TOF/PSF+TOF reconstruction with matrix size 400 × 400. [file MP-46-665-s002.tif]

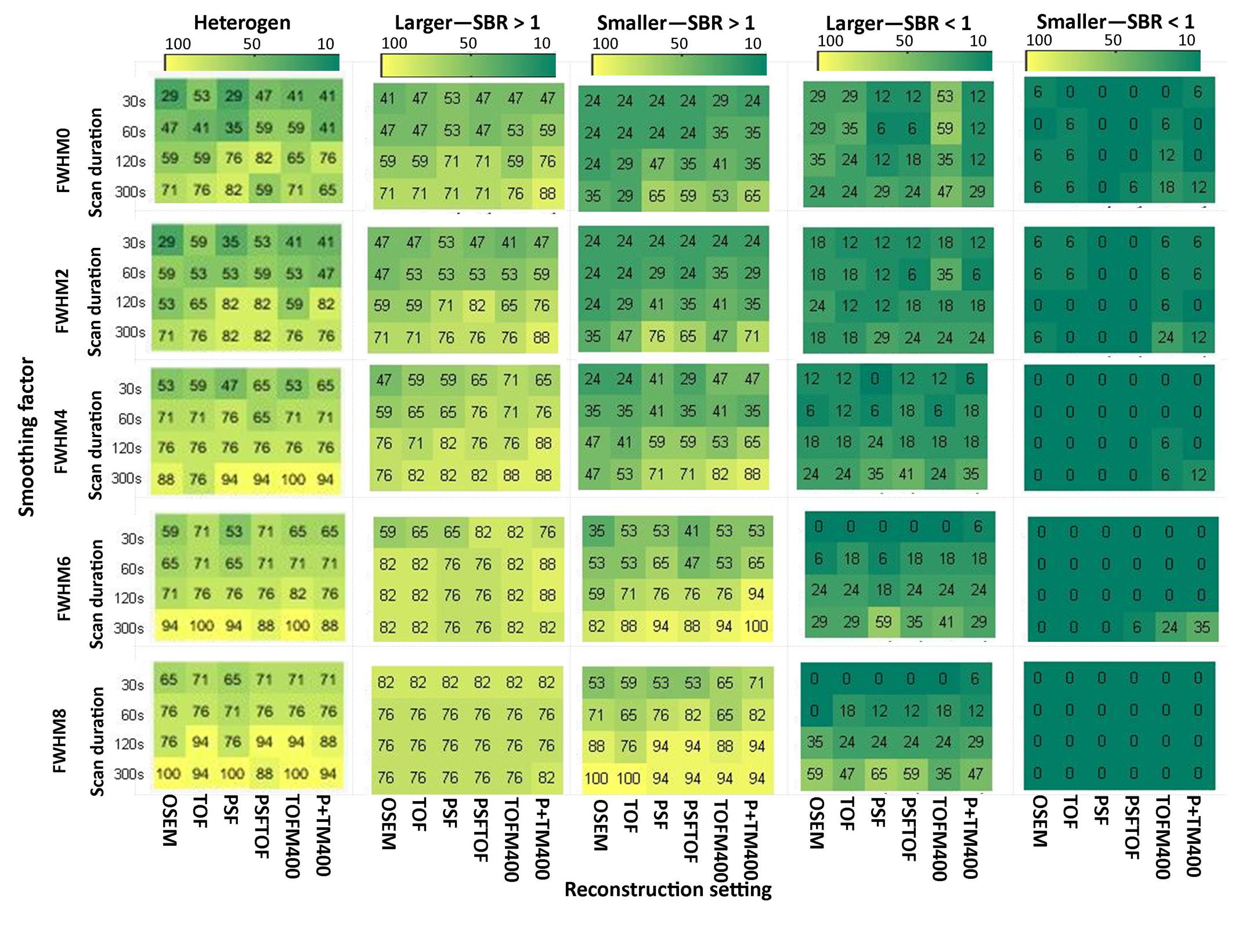

Supplement: Supplementary file 3 — Fig. S3. Percentage of repeatable features discretized with FBW: Percentage of representative features discretized with FBW and segmented based on PET exhibiting an ICC > 0.8 for all studied settings and underlying data categories (from left to right: heterogeneous 3D prints, bigger spheres with high uptake, smaller spheres with high uptake, bigger spheres with low uptake, and smaller spheres with low uptake). TOFM400/P+TM400: TOF/PSF+TOF reconstruction with matrix size 400 × 400. [file MP-46-665-s003.tif]

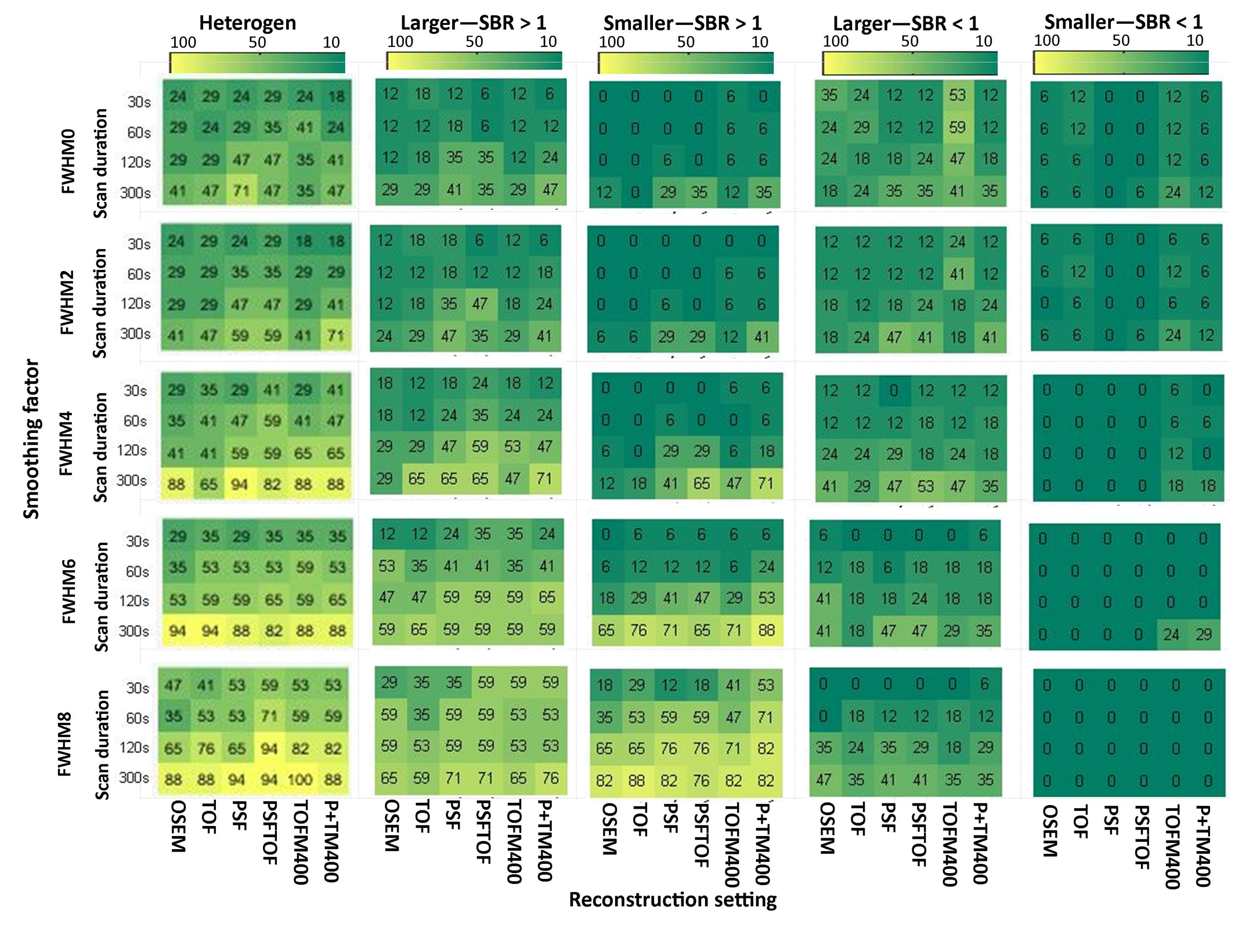

Supplement: Supplementary file 4 — Fig. S4. Percentage of repeatable features discretized with FBN: Percentage of representative features discretized with FBN and segmented based on PET exhibiting an ICC > 0.8 for all studied settings and underlying data categories (from left to right: heterogeneous 3D prints, bigger spheres with high uptake, smaller spheres with high uptake, bigger spheres with low uptake, and smaller spheres with low uptake). TOFM400/P+TM400: TOF/PSF+TOF reconstruction with matrix size 400 × 400. [file MP-46-665-s004.tif]
