# Supplementary material for: Repeatability of 18F‐FDG PET radiomic features: A phantom study to explore sensitivity to image reconstruction settings, noise, and delineation method
Source: Med Phys. 2018 Dec 28;46(2):665–78. doi: 10.1002/mp.13322 (PMC7380016; doi:10.1002/mp.13322)
Supplement: Supplementary file 5 — Table S1. ICC values for all features; extracted from EARL‐compliant reconstruction (OSEM, M256, 4 mm FWHM, CT‐based segmentation, FBW discretization). Table S2. ICC values for all features; extracted from EARL‐compliant reconstruction (OSEM, M256, 4 mm FWHM, CT‐based segmentation, FBN discretization). [file MP-46-665-s005.doc]

| Feature name | Big high uptake | Small high uptake | Big low uptake | Small low uptake | Heterogen |
| --- | --- | --- | --- | --- | --- |
| Volume | 1,00 | 1,00 | 1,00 | 1,00 | 1,00 |
| Approximate volume | 1,00 | 1,00 | 1,00 | 1,00 | 1,00 |
| Surface area | 1,00 | 1,00 | 1,00 | 1,00 | 1,00 |
| Surface to volume ratio | 1,00 | 1,00 | 1,00 | 1,00 | 1,00 |
| Compactness 1 | 1,00 | 1,00 | 1,00 | 1,00 | 1,00 |
| Compactness 2 | 1,00 | 1,00 | 1,00 | 1,00 | 1,00 |
| Spherical disproportion | 1,00 | 1,00 | 1,00 | 1,00 | 1,00 |
| Sphericity | 1,00 | 1,00 | 1,00 | 1,00 | 1,00 |
| Asphericity | 1,00 | 1,00 | 1,00 | 1,00 | 1,00 |
| Centre of mass shift | 0,31 | 0,16 | 0,21 | -0,01 | 0,99 |
| Maximum 3D diameter | 1,00 | 1,00 | 1,00 | 1,00 | 1,00 |
| Major axis length | 1,00 | 1,00 | 1,00 | 1,00 | 1,00 |
| Minor axis length | 1,00 | 1,00 | 1,00 | 1,00 | 1,00 |
| Least axis length | 1,00 | 1,00 | 1,00 | 1,00 | 1,00 |
| Elongation | 1,00 | 1,00 | 1,00 | 1,00 | 1,00 |
| Flatness | 1,00 | 1,00 | 1,00 | 1,00 | 1,00 |
| Integrated intensity | 1,00 | 1,00 | 0,99 | 0,97 | 1,00 |
| Morans I index | 0,62 | 0,44 | 0,11 | 0,37 | 1,00 |
| Gearys C measure | 0,48 | 0,24 | 0,21 | 0,11 | 0,98 |
| Local intensity peak | 0,95 | 0,97 | 0,23 | 0,07 | 0,75 |
| Local intensity dal | 0,93 | 0,92 | 0,82 | 0,36 | 0,91 |
| Global intensity peak | 0,99 | 0,99 | 0,65 | 0,21 | 0,76 |
| Mean | 1,00 | 0,98 | 0,91 | 0,40 | 1,00 |
| Variance | 0,98 | 0,91 | 0,71 | 0,17 | 0,91 |
| Skewness | 0,72 | 0,14 | 0,11 | 0,04 | 0,96 |
| Kurtosis | 0,41 | 0,02 | 0,04 | 0,09 | 0,51 |
| Median | 1,00 | 0,97 | 0,89 | 0,34 | 1,00 |
| Minimum | 0,93 | 0,89 | 0,78 | 0,47 | 0,96 |
| P10 | 0,99 | 0,96 | 0,88 | 0,40 | 1,00 |
| P90 | 0,99 | 0,97 | 0,88 | 0,31 | 0,99 |
| Maximum | 0,94 | 0,92 | 0,51 | 0,31 | 0,70 |
| Interquartile range | 0,97 | 0,91 | 0,62 | 0,32 | 0,90 |
| Range | 0,89 | 0,88 | 0,46 | 0,37 | 0,05 |
| Mean absolute deviation | 0,98 | 0,92 | 0,70 | 0,28 | 0,93 |
| Robust mean absolute deviation | 0,98 | 0,93 | 0,62 | 0,28 | 0,93 |
| Median absolute deviation | 0,98 | 0,93 | 0,70 | 0,29 | 0,92 |
| Coefficient of variation | 0,79 | 0,13 | 0,32 | 0,16 | 0,99 |
| Quartile coefficient of dispersion | 0,74 | 0,24 | 0,17 | 0,16 | 0,99 |
| Energy intensity | 1,00 | 0,99 | 0,98 | 0,89 | 1,00 |
| Root mean square | 1,00 | 0,98 | 0,90 | 0,39 | 1,00 |
| Joint maximum_2Davg_ | 0,96 | 0,95 | 0,86 | 0,82 | 0,97 |
| Joint average_2Davg_ | 0,97 | 0,95 | 0,73 | 0,42 | 0,88 |
| Joint variance_2Davg_ | 0,98 | 0,84 | 0,75 | 0,19 | 0,13 |
| Joint entropy_2Davg_ | 1,00 | 0,99 | 0,97 | 0,98 | 1,00 |
| Difference average_2Davg_ | 0,98 | 0,80 | 0,84 | 0,27 | 0,78 |
| Difference variance_2Davg_ | 0,97 | 0,77 | 0,75 | 0,22 | 0,91 |
| Difference entropy_2Davg_ | 0,98 | 0,95 | 0,90 | 0,95 | 0,99 |
| Sum average_2Davg_ | 0,97 | 0,95 | 0,73 | 0,42 | 0,88 |
| Sum variance_2Davg_ | 0,97 | 0,87 | 0,68 | 0,17 | 0,19 |
| Sum entropy_2Davg_ | 0,99 | 0,99 | 0,93 | 0,98 | 0,99 |
| Angular second moment_2Davg_ | 0,97 | 0,98 | 0,95 | 0,89 | 0,99 |
| Contrast GLCM_2Davg_ | 0,98 | 0,79 | 0,76 | 0,23 | 0,72 |
| Dissimilarity_2Davg_ | 0,98 | 0,80 | 0,84 | 0,27 | 0,78 |
| Inverse difference_2Davg_ | 0,95 | 0,78 | 0,85 | 0,19 | 0,78 |
| Inverse difference normalised_2Davg_ | 0,73 | 0,62 | 0,24 | 0,53 | 0,03 |
| Inverse difference moment_2Davg_ | 0,94 | 0,74 | 0,85 | 0,17 | 0,77 |
| Inverse difference moment normalised_2Davg_ | 0,80 | 0,54 | 0,22 | 0,63 | -0,20 |
| Inverse variance_2Davg_ | 0,92 | 0,58 | 0,84 | 0,21 | 0,85 |
| Correlation_2Davg_ | 0,76 | 0,51 | 0,61 | 0,69 | 0,53 |
| Autocorrelation_2Davg_ | 0,96 | 0,94 | 0,71 | 0,34 | 0,81 |
| Cluster tendency_2Davg_ | 0,97 | 0,87 | 0,68 | 0,17 | 0,19 |
| Cluster shade_2Davg_ | 0,92 | 0,56 | 0,49 | 0,23 | 0,28 |
| Cluster prominence_2Davg_ | 0,96 | 0,83 | 0,47 | 0,08 | 0,30 |
| First measure of information correlation_2Davg_ | 0,98 | 0,81 | 0,87 | 0,46 | 0,96 |
| Second measure of information correlation_2Davg_ | 0,91 | 0,79 | 0,75 | 0,37 | 0,16 |
| Joint maximum_2Dcomb_ | 0,84 | 0,73 | 0,61 | 0,27 | 0,96 |
| Joint average_2Dcomb_ | 0,97 | 0,95 | 0,73 | 0,42 | 0,89 |
| Joint variance_2Dcomb_ | 0,98 | 0,84 | 0,74 | 0,18 | 0,18 |
| Joint entropy_2Dcomb_ | 0,98 | 0,94 | 0,88 | 0,81 | 0,99 |
| Difference average_2Dcomb_ | 0,98 | 0,80 | 0,84 | 0,27 | 0,83 |
| Difference variance_2Dcomb_ | 0,98 | 0,77 | 0,73 | 0,20 | 0,91 |
| Difference entropy_2Dcomb_ | 0,97 | 0,87 | 0,80 | 0,56 | 0,99 |
| Sum average_2Dcomb_ | 0,97 | 0,95 | 0,73 | 0,42 | 0,89 |
| Sum variance_2Dcomb_ | 0,97 | 0,86 | 0,69 | 0,16 | 0,10 |
| Sum entropy_2Dcomb_ | 0,97 | 0,92 | 0,76 | 0,77 | 0,97 |
| Angular second moment_2Dcomb_ | 0,86 | 0,89 | 0,70 | 0,42 | 0,99 |
| Contrast GLCM_2Dcomb_ | 0,98 | 0,79 | 0,76 | 0,23 | 0,78 |
| Dissimilarity_2Dcomb_ | 0,98 | 0,80 | 0,84 | 0,27 | 0,83 |
| Inverse difference_2Dcomb_ | 0,95 | 0,78 | 0,85 | 0,20 | 0,79 |
| Inverse difference normalised_2Dcomb_ | 0,73 | 0,61 | 0,24 | 0,54 | 0,18 |
| Inverse difference moment_2Dcomb_ | 0,94 | 0,75 | 0,85 | 0,18 | 0,79 |
| Inverse difference moment normalised_2Dcomb_ | 0,80 | 0,54 | 0,22 | 0,65 | -0,13 |
| Inverse variance_2Dcomb_ | 0,93 | 0,58 | 0,84 | 0,22 | 0,92 |
| Correlation_2Dcomb_ | 0,72 | 0,35 | 0,55 | 0,62 | -0,03 |
| Autocorrelation_2Dcomb_ | 0,96 | 0,94 | 0,71 | 0,34 | 0,83 |
| Cluster tendency_2Dcomb_ | 0,97 | 0,86 | 0,69 | 0,16 | 0,10 |
| Cluster shade_2Dcomb_ | 0,92 | 0,57 | 0,51 | 0,14 | 0,31 |
| Cluster prominence_2Dcomb_ | 0,96 | 0,82 | 0,48 | 0,06 | 0,24 |
| First measure of information correlation_2Dcomb_ | 0,96 | 0,69 | 0,68 | 0,04 | 0,94 |
| Second measure of information correlation_2Dcomb_ | 0,94 | 0,75 | 0,61 | 0,25 | 0,12 |
| Joint maximum_3Davg_ | 0,92 | 0,91 | 0,63 | 0,77 | 0,46 |
| Joint average_3Davg_ | 0,98 | 0,96 | 0,73 | 0,42 | 0,84 |
| Joint variance_3Davg_ | 0,97 | 0,90 | 0,71 | 0,19 | 0,93 |
| Joint entropy_3Davg_ | 0,98 | 0,99 | 0,82 | 0,97 | 0,57 |
| Difference average_3Davg_ | 0,98 | 0,88 | 0,86 | 0,27 | 0,62 |
| Difference variance_3Davg_ | 0,97 | 0,82 | 0,79 | 0,23 | 0,62 |
| Difference entropy_3Davg_ | 0,98 | 0,94 | 0,86 | 0,81 | 0,70 |
| Sum average_3Davg_ | 0,98 | 0,96 | 0,73 | 0,42 | 0,84 |
| Sum variance_3Davg_ | 0,97 | 0,91 | 0,62 | 0,18 | 0,95 |
| Sum entropy_3Davg_ | 0,97 | 0,97 | 0,67 | 0,95 | 0,95 |
| Angular second moment_3Davg_ | 0,97 | 0,97 | 0,70 | 0,93 | 0,72 |
| Contrast GLCM_3Davg_ | 0,97 | 0,85 | 0,82 | 0,23 | 0,58 |
| Dissimilarity_3Davg_ | 0,98 | 0,88 | 0,86 | 0,27 | 0,62 |
| Inverse difference_3Davg_ | 0,98 | 0,87 | 0,86 | 0,29 | 0,70 |
| Inverse difference normalised_3Davg_ | 0,79 | 0,62 | 0,29 | 0,60 | -0,23 |
| Inverse difference moment_3Davg_ | 0,98 | 0,87 | 0,86 | 0,27 | 0,72 |
| Inverse difference moment normalised_3Davg_ | 0,82 | 0,56 | 0,28 | 0,66 | -0,14 |
| Inverse variance_3Davg_ | 0,97 | 0,82 | 0,87 | 0,13 | 0,72 |
| Correlation_3Davg_ | 0,52 | 0,37 | 0,44 | 0,50 | 0,99 |
| Autocorrelation_3Davg_ | 0,97 | 0,96 | 0,70 | 0,34 | 0,75 |
| Cluster tendency_3Davg_ | 0,97 | 0,91 | 0,62 | 0,18 | 0,95 |
| Cluster shade_3Davg_ | 0,87 | 0,01 | 0,49 | 0,09 | 0,90 |
| Cluster prominence_3Davg_ | 0,95 | 0,81 | 0,48 | 0,05 | 0,87 |
| First measure of information correlation_3Davg_ | 0,98 | 0,89 | 0,78 | 0,84 | 0,99 |
| Second measure of information correlation_3Davg_ | 0,98 | 0,81 | 0,77 | 0,62 | 0,99 |
| Joint maximum_3Dcomb_ | 0,90 | 0,66 | 0,54 | 0,31 | 0,21 |
| Joint average_3Dcomb_ | 0,98 | 0,96 | 0,73 | 0,42 | 0,84 |
| Joint variance_3Dcomb_ | 0,97 | 0,90 | 0,71 | 0,19 | 0,93 |
| Joint entropy_3Dcomb_ | 0,98 | 0,94 | 0,78 | 0,70 | 0,75 |
| Difference average_3Dcomb_ | 0,98 | 0,89 | 0,86 | 0,28 | 0,63 |
| Difference variance_3Dcomb_ | 0,97 | 0,83 | 0,79 | 0,20 | 0,55 |
| Difference entropy_3Dcomb_ | 0,98 | 0,86 | 0,86 | 0,32 | 0,58 |
| Sum average_3Dcomb_ | 0,98 | 0,96 | 0,73 | 0,42 | 0,84 |
| Sum variance_3Dcomb_ | 0,97 | 0,91 | 0,62 | 0,17 | 0,95 |
| Sum entropy_3Dcomb_ | 0,97 | 0,90 | 0,67 | 0,49 | 0,95 |
| Angular second moment_3Dcomb_ | 0,96 | 0,81 | 0,70 | 0,52 | 0,81 |
| Contrast GLCM_3Dcomb_ | 0,97 | 0,86 | 0,82 | 0,23 | 0,59 |
| Dissimilarity_3Dcomb_ | 0,98 | 0,89 | 0,86 | 0,28 | 0,63 |
| Inverse difference_3Dcomb_ | 0,98 | 0,88 | 0,86 | 0,30 | 0,72 |
| Inverse difference normalised_3Dcomb_ | 0,78 | 0,59 | 0,29 | 0,58 | -0,24 |
| Inverse difference moment_3Dcomb_ | 0,98 | 0,87 | 0,86 | 0,28 | 0,74 |
| Inverse difference moment normalised_3Dcomb_ | 0,82 | 0,54 | 0,28 | 0,65 | -0,17 |
| Inverse variance_3Dcomb_ | 0,97 | 0,83 | 0,87 | 0,14 | 0,75 |
| Correlation_3Dcomb_ | 0,52 | 0,37 | 0,44 | 0,49 | 0,99 |
| Autocorrelation_3Dcomb_ | 0,97 | 0,96 | 0,70 | 0,34 | 0,75 |
| Cluster tendency_3Dcomb_ | 0,97 | 0,91 | 0,62 | 0,17 | 0,95 |
| Cluster shade_3Dcomb_ | 0,87 | 0,02 | 0,50 | 0,06 | 0,90 |
| Cluster prominence_3Dcomb_ | 0,95 | 0,81 | 0,48 | 0,04 | 0,87 |
| First measure of information correlation_3Dcomb_ | 0,28 | 0,63 | 0,27 | 0,08 | 1,00 |
| Second measure of information correlation_3Dcomb_ | 0,65 | 0,72 | 0,09 | 0,16 | 1,00 |
| Short runs emphasis_2Davg_ | 0,90 | 0,49 | 0,78 | 0,24 | 0,59 |
| Long runs emphasis_2Davg_ | 0,91 | 0,48 | 0,79 | 0,22 | 0,58 |
| Low grey level run emphasis_2Davg_ | 0,81 | 0,49 | 0,55 | 0,51 | 0,61 |
| High grey level run emphasis_2Davg_ | 0,96 | 0,95 | 0,75 | 0,35 | 0,84 |
| Short run low grey level emphasis_2Davg_ | 0,80 | 0,49 | 0,50 | 0,50 | 0,60 |
| Short run high grey level emphasis_2Davg_ | 0,96 | 0,95 | 0,75 | 0,34 | 0,84 |
| Long run low grey level emphasis_2Davg_ | 0,79 | 0,47 | 0,61 | 0,47 | 0,66 |
| Long run high grey level emphasis_2Davg_ | 0,96 | 0,95 | 0,73 | 0,39 | 0,85 |
| Grey level non uniformity GLRLM_2Davg_ | 0,99 | 0,87 | 0,98 | 0,85 | 0,99 |
| Grey level non uniformity normalised GLRLM_2Davg_ | 0,94 | 0,99 | 0,78 | 0,94 | 0,97 |
| Run length non uniformity_2Davg_ | 1,00 | 0,99 | 1,00 | 0,99 | 1,00 |
| Run length non uniformity normalised_2Davg_ | 0,92 | 0,55 | 0,78 | 0,37 | 0,60 |
| Run percentage_2Davg_ | 0,92 | 0,54 | 0,79 | 0,32 | 0,59 |
| Grey level variance GLRLM_2Davg_ | 0,98 | 0,91 | 0,76 | 0,20 | 0,58 |
| Run length variance_2Davg_ | 0,92 | 0,49 | 0,79 | 0,32 | 0,05 |
| Run entropy_2Davg_ | 0,98 | 0,99 | 0,92 | 0,96 | 0,99 |
| Short runs emphasis_2Dcomb_ | 0,91 | 0,52 | 0,78 | 0,26 | 0,59 |
| Long runs emphasis_2Dcomb_ | 0,92 | 0,51 | 0,80 | 0,25 | 0,57 |
| Low grey level run emphasis_2Dcomb_ | 0,81 | 0,49 | 0,55 | 0,51 | 0,61 |
| High grey level run emphasis_2Dcomb_ | 0,96 | 0,95 | 0,75 | 0,35 | 0,84 |
| Short run low grey level emphasis_2Dcomb_ | 0,80 | 0,49 | 0,50 | 0,50 | 0,60 |
| Short run high grey level emphasis_2Dcomb_ | 0,96 | 0,95 | 0,75 | 0,34 | 0,84 |
| Long run low grey level emphasis_2Dcomb_ | 0,79 | 0,47 | 0,60 | 0,48 | 0,66 |
| Long run high grey level emphasis_2Dcomb_ | 0,96 | 0,95 | 0,73 | 0,39 | 0,85 |
| Grey level non uniformity GLRLM_2Dcomb_ | 0,99 | 0,87 | 0,98 | 0,85 | 0,99 |
| Grey level non uniformity normalised GLRLM_2Dcomb_ | 0,95 | 0,99 | 0,78 | 0,94 | 0,97 |
| Run length non uniformity_2Dcomb_ | 1,00 | 0,99 | 1,00 | 0,99 | 1,00 |
| Run length non uniformity normalised_2Dcomb_ | 0,92 | 0,53 | 0,78 | 0,32 | 0,59 |
| Run percentage_2Dcomb_ | 0,92 | 0,54 | 0,79 | 0,32 | 0,59 |
| Grey level variance GLRLM_2Dcomb_ | 0,98 | 0,91 | 0,76 | 0,20 | 0,58 |
| Run length variance_2Dcomb_ | 0,92 | 0,49 | 0,79 | 0,30 | 0,06 |
| Run entropy_2Dcomb_ | 0,99 | 0,99 | 0,91 | 0,98 | 1,00 |
| Short runs emphasis_3Davg_ | 0,98 | 0,76 | 0,84 | 0,47 | 0,74 |
| Long runs emphasis_3Davg_ | 0,97 | 0,72 | 0,85 | 0,43 | 0,79 |
| Low grey level run emphasis_3Davg_ | 0,84 | 0,69 | 0,53 | 0,61 | 0,84 |
| High grey level run emphasis_3Davg_ | 0,97 | 0,95 | 0,73 | 0,35 | 0,80 |
| Short run low grey level emphasis_3Davg_ | 0,83 | 0,71 | 0,49 | 0,64 | 0,83 |
| Short run high grey level emphasis_3Davg_ | 0,97 | 0,95 | 0,74 | 0,34 | 0,80 |
| Long run low grey level emphasis_3Davg_ | 0,87 | 0,60 | 0,60 | 0,49 | 0,86 |
| Long run high grey level emphasis_3Davg_ | 0,97 | 0,96 | 0,71 | 0,38 | 0,81 |
| Grey level non uniformity GLRLM_3Davg_ | 1,00 | 0,95 | 0,99 | 0,95 | 1,00 |
| Grey level non uniformity normalised GLRLM_3Davg_ | 0,98 | 0,87 | 0,65 | 0,63 | 0,86 |
| Run length non uniformity_3Davg_ | 1,00 | 1,00 | 1,00 | 1,00 | 1,00 |
| Run length non uniformity normalised_3Davg_ | 0,98 | 0,76 | 0,84 | 0,50 | 0,73 |
| Run percentage_3Davg_ | 0,97 | 0,75 | 0,85 | 0,48 | 0,76 |
| Grey level variance GLRLM_3Davg_ | 0,98 | 0,92 | 0,72 | 0,16 | 0,91 |
| Run length variance_3Davg_ | 0,96 | 0,68 | 0,85 | 0,42 | 0,81 |
| Run entropy_3Davg_ | 0,98 | 0,95 | 0,69 | 0,82 | 0,90 |
| Short runs emphasis_3Dcomb_ | 0,98 | 0,75 | 0,84 | 0,49 | 0,73 |
| Long runs emphasis_3Dcomb_ | 0,97 | 0,73 | 0,85 | 0,45 | 0,78 |
| Low grey level run emphasis_3Dcomb_ | 0,84 | 0,69 | 0,53 | 0,61 | 0,84 |
| High grey level run emphasis_3Dcomb_ | 0,97 | 0,95 | 0,73 | 0,35 | 0,80 |
| Short run low grey level emphasis_3Dcomb_ | 0,83 | 0,71 | 0,49 | 0,64 | 0,83 |
| Short run high grey level emphasis_3Dcomb_ | 0,97 | 0,95 | 0,74 | 0,34 | 0,80 |
| Long run low grey level emphasis_3Dcomb_ | 0,87 | 0,60 | 0,60 | 0,49 | 0,86 |
| Long run high grey level emphasis_3Dcomb_ | 0,97 | 0,96 | 0,71 | 0,38 | 0,81 |
| Grey level non uniformity GLRLM_3Dcomb_ | 1,00 | 0,95 | 0,99 | 0,95 | 1,00 |
| Grey level non uniformity normalised GLRLM_3Dcomb_ | 0,98 | 0,87 | 0,65 | 0,62 | 0,86 |
| Run length non uniformity_3Dcomb_ | 1,00 | 1,00 | 1,00 | 1,00 | 1,00 |
| Run length non uniformity normalised_3Dcomb_ | 0,98 | 0,76 | 0,84 | 0,49 | 0,73 |
| Run percentage_3Dcomb_ | 0,97 | 0,75 | 0,85 | 0,48 | 0,76 |
| Grey level variance GLRLM_3Dcomb_ | 0,98 | 0,91 | 0,72 | 0,16 | 0,91 |
| Run length variance_3Dcomb_ | 0,96 | 0,69 | 0,85 | 0,42 | 0,80 |
| Run entropy_3Dcomb_ | 0,98 | 0,95 | 0,66 | 0,81 | 0,92 |
| Small zone emphasis_2D_ | 0,84 | 0,45 | 0,73 | 0,25 | 0,51 |
| Large zone emphasis_2D_ | 0,91 | 0,47 | 0,76 | 0,28 | 0,54 |
| Low grey level zone emphasis_2D_ | 0,80 | 0,52 | 0,45 | 0,50 | 0,62 |
| High grey level zone emphasis_2D_ | 0,96 | 0,95 | 0,74 | 0,37 | 0,85 |
| Small zone low grey level emphasis_2D_ | 0,67 | 0,48 | 0,23 | 0,45 | 0,58 |
| Small zone high grey level emphasis_2D_ | 0,97 | 0,93 | 0,75 | 0,33 | 0,85 |
| Large zone low grey level emphasis_2D_ | 0,58 | 0,39 | 0,62 | 0,20 | 0,80 |
| Large zone high grey level emphasis_2D_ | 0,92 | 0,92 | 0,60 | 0,53 | 0,86 |
| Grey level non uniformity GLSZM_2D_ | 0,99 | 0,80 | 0,99 | 0,86 | 0,99 |
| Grey level non uniformity normalised GLSZM_2D_ | 0,93 | 0,99 | 0,82 | 0,95 | 0,98 |
| Zone size non uniformity_2D_ | 0,98 | 0,94 | 0,96 | 0,85 | 0,99 |
| Zone size non uniformity normalised_2D_ | 0,91 | 0,60 | 0,77 | 0,51 | 0,49 |
| Zone percentage_2D_ | 0,92 | 0,55 | 0,78 | 0,43 | 0,55 |
| Grey level variance GLSZM_2D_ | 0,98 | 0,91 | 0,76 | 0,20 | 0,65 |
| Zone size variance_2D_ | 0,89 | 0,47 | 0,75 | 0,45 | 0,02 |
| Zone size entropy_2D_ | 0,98 | 0,98 | 0,96 | 0,95 | 1,00 |
| Small zone emphasis_3D_ | 0,76 | 0,60 | 0,19 | 0,12 | 0,52 |
| Large zone emphasis_3D_ | 0,89 | 0,67 | 0,93 | 0,53 | 0,79 |
| Low grey level zone emphasis_3D_ | 0,79 | 0,70 | 0,19 | 0,73 | 0,75 |
| High grey level zone emphasis_3D_ | 0,96 | 0,92 | 0,71 | 0,39 | 0,77 |
| Small zone low grey level emphasis_3D_ | 0,51 | 0,11 | 0,00 | 0,39 | 0,61 |
| Small zone high grey level emphasis_3D_ | 0,94 | 0,86 | 0,69 | 0,27 | 0,82 |
| Large zone low grey level emphasis_3D_ | 0,92 | 0,31 | 0,88 | 0,26 | 0,91 |
| Large zone high grey level emphasis_3D_ | 0,85 | 0,72 | 0,88 | 0,55 | 0,89 |
| Grey level non uniformity GLSZM_3D_ | 0,99 | 0,91 | 0,97 | 0,91 | 1,00 |
| Grey level non uniformity normalised GLSZM_3D_ | 0,97 | 0,83 | 0,75 | 0,60 | 0,52 |
| Zone size non uniformity_3D_ | 0,99 | 0,94 | 0,95 | 0,70 | 0,99 |
| Zone size non uniformity normalised_3D_ | 0,84 | 0,65 | 0,15 | 0,12 | 0,54 |
| Zone percentage_3D_ | 0,97 | 0,78 | 0,87 | 0,54 | 0,76 |
| Grey level variance GLSZM_3D_ | 0,96 | 0,89 | 0,71 | 0,17 | 0,61 |
| Zone size variance_3D_ | 0,88 | 0,65 | 0,93 | 0,48 | 0,78 |
| Zone size entropy_3D_ | 0,95 | 0,92 | 0,81 | 0,90 | 0,33 |
| Coarseness_2D_ | 0,88 | 0,72 | 0,83 | 0,44 | 0,98 |
| Contrast_2D_ | 0,93 | 0,28 | 0,31 | 0,35 | 0,28 |
| Busyness_2D_ | 0,48 | 0,37 | 0,07 | 0,13 | 0,50 |
| Complexity_2D_ | 0,98 | 0,89 | 0,82 | 0,29 | 0,92 |
| Texture strength_2D_ | 0,96 | 0,79 | 0,67 | 0,18 | 0,79 |
| Coarseness_3D_ | 0,99 | 0,83 | 0,99 | 0,81 | 1,00 |
| Contrast_3D_ | 0,95 | 0,53 | 0,86 | 0,36 | 0,91 |
| Busyness_3D_ | 0,91 | 0,42 | 0,83 | 0,21 | 0,51 |
| Complexity_3D_ | 0,95 | 0,91 | 0,70 | 0,29 | 0,30 |
| Texture strength_3D_ | 0,92 | 0,72 | 0,64 | 0,12 | 0,86 |

Table S-1: ICC values for all features; extracted from EARL-compliant reconstruction

(OSEM, M256, 4 mm FWHM, CT-based segmentation, FBW discretization)

| Feature name | Big hot | Small hot | Big cold | Small cold |  |
| --- | --- | --- | --- | --- | --- |
| Volume | 1,00 | 1,00 | 1,00 | 1,00 | 1,00 |
| Approximate volume | 1,00 | 1,00 | 1,00 | 1,00 | 1,00 |
| Surface area | 1,00 | 1,00 | 1,00 | 1,00 | 1,00 |
| Surface to volume ratio | 1,00 | 1,00 | 1,00 | 1,00 | 1,00 |
| Compactness 1 | 1,00 | 1,00 | 1,00 | 1,00 | 1,00 |
| Compactness 2 | 1,00 | 1,00 | 1,00 | 1,00 | 1,00 |
| Spherical disproportion | 1,00 | 1,00 | 1,00 | 1,00 | 1,00 |
| Sphericity | 1,00 | 1,00 | 1,00 | 1,00 | 1,00 |
| Asphericity | 1,00 | 1,00 | 1,00 | 1,00 | 1,00 |
| Centre of mass shift | 0,31 | 0,16 | 0,21 | -0,01 | 0,99 |
| Maximum 3D diameter | 1,00 | 1,00 | 1,00 | 1,00 | 1,00 |
| Major axis length | 1,00 | 1,00 | 1,00 | 1,00 | 1,00 |
| Minor axis length | 1,00 | 1,00 | 1,00 | 1,00 | 1,00 |
| Least axis length | 1,00 | 1,00 | 1,00 | 1,00 | 1,00 |
| Elongation | 1,00 | 1,00 | 1,00 | 1,00 | 1,00 |
| Flatness | 1,00 | 1,00 | 1,00 | 1,00 | 1,00 |
| Integrated intensity | 1,00 | 1,00 | 0,99 | 0,97 | 1,00 |
| Morans I index | 0,62 | 0,44 | 0,11 | 0,37 | 1,00 |
| Gearys C measure | 0,48 | 0,24 | 0,21 | 0,11 | 0,98 |
| Local intensity peak | 0,95 | 0,97 | 0,23 | 0,07 | 0,75 |
| Local intensity dal | 0,93 | 0,92 | 0,82 | 0,36 | 0,91 |
| Global intensity peak | 0,99 | 0,99 | 0,65 | 0,21 | 0,76 |
| Mean | 1,00 | 0,98 | 0,91 | 0,40 | 1,00 |
| Variance | 0,98 | 0,91 | 0,71 | 0,17 | 0,91 |
| Skewness | 0,72 | 0,14 | 0,11 | 0,04 | 0,96 |
| Kurtosis | 0,41 | 0,02 | 0,04 | 0,09 | 0,51 |
| Median | 1,00 | 0,97 | 0,89 | 0,34 | 1,00 |
| Minimum | 0,93 | 0,89 | 0,78 | 0,47 | 0,96 |
| P10 | 0,99 | 0,96 | 0,88 | 0,40 | 1,00 |
| P90 | 0,99 | 0,97 | 0,88 | 0,31 | 0,99 |
| Maximum | 0,94 | 0,92 | 0,51 | 0,31 | 0,70 |
| Interquartile range | 0,97 | 0,91 | 0,62 | 0,32 | 0,90 |
| Range | 0,89 | 0,88 | 0,46 | 0,37 | 0,05 |
| Mean absolute deviation | 0,98 | 0,92 | 0,70 | 0,28 | 0,93 |
| Robust mean absolute deviation | 0,98 | 0,93 | 0,62 | 0,28 | 0,93 |
| Median absolute deviation | 0,98 | 0,93 | 0,70 | 0,29 | 0,92 |
| Coefficient of variation | 0,79 | 0,13 | 0,32 | 0,16 | 0,99 |
| Quartile coefficient of dispersion | 0,74 | 0,24 | 0,17 | 0,16 | 0,99 |
| Energy intensity | 1,00 | 0,99 | 0,98 | 0,89 | 1,00 |
| Root mean square | 1,00 | 0,98 | 0,90 | 0,39 | 1,00 |
| Joint maximum_2Davg_ | 0,96 | 0,95 | 0,92 | 0,96 | 0,98 |
| Joint average_2Davg_ | 0,51 | 0,26 | 0,03 | 0,11 | 0,82 |
| Joint variance_2Davg_ | 0,76 | 0,51 | 0,20 | 0,45 | -0,02 |
| Joint entropy_2Davg_ | 1,00 | 1,00 | 0,99 | 1,00 | 1,00 |
| Difference average_2Davg_ | 0,75 | 0,63 | 0,24 | 0,62 | -0,05 |
| Difference variance_2Davg_ | 0,75 | 0,16 | 0,19 | 0,23 | -0,07 |
| Difference entropy_2Davg_ | 0,88 | 0,99 | 0,81 | 0,99 | 0,94 |
| Sum average_2Davg_ | 0,51 | 0,26 | 0,03 | 0,11 | 0,82 |
| Sum variance_2Davg_ | 0,72 | 0,31 | 0,23 | 0,04 | 0,22 |
| Sum entropy_2Davg_ | 0,98 | 1,00 | 0,96 | 1,00 | 0,98 |
| Angular second moment_2Davg_ | 0,98 | 0,99 | 0,98 | 0,99 | 0,99 |
| Contrast GLCM_2Davg_ | 0,80 | 0,55 | 0,23 | 0,66 | -0,22 |
| Dissimilarity_2Davg_ | 0,75 | 0,63 | 0,24 | 0,62 | -0,05 |
| Inverse difference_2Davg_ | 0,56 | 0,50 | 0,22 | 0,34 | 0,45 |
| Inverse difference normalised_2Davg_ | 0,72 | 0,66 | 0,24 | 0,58 | 0,04 |
| Inverse difference moment_2Davg_ | 0,49 | 0,32 | 0,20 | 0,25 | 0,50 |
| Inverse difference moment normalised_2Davg_ | 0,79 | 0,59 | 0,23 | 0,64 | -0,20 |
| Inverse variance_2Davg_ | 0,38 | 0,34 | 0,20 | 0,23 | 0,53 |
| Correlation_2Davg_ | 0,76 | 0,51 | 0,62 | 0,70 | 0,50 |
| Autocorrelation_2Davg_ | 0,52 | 0,25 | 0,05 | 0,05 | 0,70 |
| Cluster tendency_2Davg_ | 0,72 | 0,31 | 0,23 | 0,04 | 0,22 |
| Cluster shade_2Davg_ | 0,83 | 0,08 | 0,24 | 0,15 | 0,22 |
| Cluster prominence_2Davg_ | 0,75 | 0,20 | 0,25 | -0,11 | 0,44 |
| First measure of information correlation_2Davg_ | 0,90 | 0,51 | 0,77 | 0,58 | 0,92 |
| Second measure of information correlation_2Davg_ | 0,60 | 0,62 | 0,33 | 0,60 | 0,38 |
| Joint maximum_2Dcomb_ | 0,57 | 0,55 | 0,48 | 0,43 | 0,98 |
| Joint average_2Dcomb_ | 0,51 | 0,25 | 0,03 | 0,11 | 0,86 |
| Joint variance_2Dcomb_ | 0,76 | 0,54 | 0,19 | 0,51 | -0,10 |
| Joint entropy_2Dcomb_ | 0,96 | 0,97 | 0,93 | 0,97 | 0,99 |
| Difference average_2Dcomb_ | 0,75 | 0,63 | 0,24 | 0,63 | 0,09 |
| Difference variance_2Dcomb_ | 0,78 | 0,37 | 0,17 | 0,46 | -0,02 |
| Difference entropy_2Dcomb_ | 0,58 | 0,92 | 0,38 | 0,87 | 0,85 |
| Sum average_2Dcomb_ | 0,51 | 0,25 | 0,03 | 0,11 | 0,86 |
| Sum variance_2Dcomb_ | 0,72 | 0,41 | 0,21 | 0,15 | 0,13 |
| Sum entropy_2Dcomb_ | 0,79 | 0,97 | 0,69 | 0,94 | 0,90 |
| Angular second moment_2Dcomb_ | 0,85 | 0,87 | 0,76 | 0,84 | 0,98 |
| Contrast GLCM_2Dcomb_ | 0,80 | 0,56 | 0,23 | 0,68 | -0,16 |
| Dissimilarity_2Dcomb_ | 0,75 | 0,63 | 0,24 | 0,63 | 0,09 |
| Inverse difference_2Dcomb_ | 0,56 | 0,47 | 0,22 | 0,34 | 0,50 |
| Inverse difference normalised_2Dcomb_ | 0,72 | 0,65 | 0,24 | 0,59 | 0,19 |
| Inverse difference moment_2Dcomb_ | 0,49 | 0,30 | 0,20 | 0,26 | 0,51 |
| Inverse difference moment normalised_2Dcomb_ | 0,79 | 0,59 | 0,23 | 0,66 | -0,13 |
| Inverse variance_2Dcomb_ | 0,38 | 0,34 | 0,21 | 0,24 | 0,53 |
| Correlation_2Dcomb_ | 0,72 | 0,35 | 0,56 | 0,61 | -0,09 |
| Autocorrelation_2Dcomb_ | 0,52 | 0,25 | 0,05 | 0,05 | 0,74 |
| Cluster tendency_2Dcomb_ | 0,72 | 0,41 | 0,21 | 0,15 | 0,13 |
| Cluster shade_2Dcomb_ | 0,83 | -0,01 | 0,23 | -0,04 | 0,25 |
| Cluster prominence_2Dcomb_ | 0,75 | 0,25 | 0,25 | -0,06 | 0,39 |
| First measure of information correlation_2Dcomb_ | 0,78 | 0,76 | 0,49 | 0,23 | 0,92 |
| Second measure of information correlation_2Dcomb_ | 0,49 | 0,71 | 0,35 | 0,57 | -0,02 |
| Joint maximum_3Davg_ | 0,71 | 0,98 | 0,55 | 0,95 | 0,41 |
| Joint average_3Davg_ | 0,50 | 0,21 | 0,09 | 0,12 | 0,69 |
| Joint variance_3Davg_ | 0,76 | 0,52 | 0,19 | 0,50 | 0,85 |
| Joint entropy_3Davg_ | 0,91 | 1,00 | 0,81 | 1,00 | -0,04 |
| Difference average_3Davg_ | 0,79 | 0,62 | 0,30 | 0,65 | -0,23 |
| Difference variance_3Davg_ | 0,80 | 0,26 | 0,31 | 0,58 | -0,01 |
| Difference entropy_3Davg_ | 0,61 | 0,99 | 0,18 | 0,95 | -0,24 |
| Sum average_3Davg_ | 0,50 | 0,21 | 0,09 | 0,12 | 0,69 |
| Sum variance_3Davg_ | 0,71 | 0,27 | 0,15 | 0,18 | 0,91 |
| Sum entropy_3Davg_ | 0,30 | 1,00 | 0,19 | 0,98 | 0,84 |
| Angular second moment_3Davg_ | 0,89 | 1,00 | 0,59 | 1,00 | -0,22 |
| Contrast GLCM_3Davg_ | 0,82 | 0,57 | 0,30 | 0,69 | -0,14 |
| Dissimilarity_3Davg_ | 0,79 | 0,62 | 0,30 | 0,65 | -0,23 |
| Inverse difference_3Davg_ | 0,67 | 0,44 | 0,34 | 0,47 | -0,16 |
| Inverse difference normalised_3Davg_ | 0,78 | 0,63 | 0,30 | 0,62 | -0,24 |
| Inverse difference moment_3Davg_ | 0,63 | 0,30 | 0,35 | 0,41 | -0,05 |
| Inverse difference moment normalised_3Davg_ | 0,82 | 0,60 | 0,29 | 0,67 | -0,16 |
| Inverse variance_3Davg_ | 0,63 | 0,43 | 0,33 | 0,40 | 0,04 |
| Correlation_3Davg_ | 0,51 | 0,35 | 0,45 | 0,51 | 0,99 |
| Autocorrelation_3Davg_ | 0,53 | 0,23 | 0,09 | 0,07 | 0,56 |
| Cluster tendency_3Davg_ | 0,71 | 0,27 | 0,15 | 0,18 | 0,91 |
| Cluster shade_3Davg_ | 0,59 | 0,16 | 0,06 | -0,05 | 0,93 |
| Cluster prominence_3Davg_ | 0,70 | 0,33 | 0,16 | 0,12 | 0,89 |
| First measure of information correlation_3Davg_ | 0,98 | 0,94 | 0,84 | 0,88 | 0,98 |
| Second measure of information correlation_3Davg_ | 0,87 | 0,40 | 0,82 | 0,15 | 0,96 |
| Joint maximum_3Dcomb_ | -0,06 | 0,71 | 0,21 | 0,43 | 0,08 |
| Joint average_3Dcomb_ | 0,50 | 0,19 | 0,09 | 0,12 | 0,69 |
| Joint variance_3Dcomb_ | 0,76 | 0,52 | 0,19 | 0,51 | 0,85 |
| Joint entropy_3Dcomb_ | 0,27 | 0,97 | 0,16 | 0,87 | 0,39 |
| Difference average_3Dcomb_ | 0,79 | 0,59 | 0,29 | 0,64 | -0,24 |
| Difference variance_3Dcomb_ | 0,81 | 0,45 | 0,31 | 0,68 | 0,01 |
| Difference entropy_3Dcomb_ | 0,73 | 0,20 | 0,26 | 0,22 | -0,21 |
| Sum average_3Dcomb_ | 0,50 | 0,19 | 0,09 | 0,12 | 0,69 |
| Sum variance_3Dcomb_ | 0,72 | 0,35 | 0,16 | 0,25 | 0,91 |
| Sum entropy_3Dcomb_ | 0,60 | 0,80 | 0,12 | 0,35 | 0,87 |
| Angular second moment_3Dcomb_ | 0,25 | 0,90 | 0,13 | 0,75 | 0,45 |
| Contrast GLCM_3Dcomb_ | 0,82 | 0,54 | 0,29 | 0,68 | -0,17 |
| Dissimilarity_3Dcomb_ | 0,79 | 0,59 | 0,29 | 0,64 | -0,24 |
| Inverse difference_3Dcomb_ | 0,66 | 0,41 | 0,33 | 0,44 | -0,12 |
| Inverse difference normalised_3Dcomb_ | 0,77 | 0,60 | 0,29 | 0,60 | -0,25 |
| Inverse difference moment_3Dcomb_ | 0,62 | 0,29 | 0,34 | 0,38 | -0,01 |
| Inverse difference moment normalised_3Dcomb_ | 0,81 | 0,57 | 0,28 | 0,66 | -0,19 |
| Inverse variance_3Dcomb_ | 0,61 | 0,42 | 0,32 | 0,36 | 0,10 |
| Correlation_3Dcomb_ | 0,51 | 0,35 | 0,44 | 0,50 | 0,99 |
| Autocorrelation_3Dcomb_ | 0,53 | 0,21 | 0,09 | 0,08 | 0,56 |
| Cluster tendency_3Dcomb_ | 0,72 | 0,35 | 0,16 | 0,25 | 0,91 |
| Cluster shade_3Dcomb_ | 0,60 | 0,21 | 0,07 | -0,05 | 0,93 |
| Cluster prominence_3Dcomb_ | 0,71 | 0,42 | 0,17 | 0,18 | 0,89 |
| First measure of information correlation_3Dcomb_ | 0,93 | 0,56 | 0,41 | 0,37 | 1,00 |
| Second measure of information correlation_3Dcomb_ | 0,93 | 0,52 | 0,31 | 0,17 | 1,00 |
| Short runs emphasis_2Davg_ | 0,57 | 0,30 | 0,26 | 0,29 | 0,17 |
| Long runs emphasis_2Davg_ | 0,57 | 0,26 | 0,29 | 0,32 | 0,18 |
| Low grey level run emphasis_2Davg_ | 0,39 | 0,14 | 0,04 | 0,27 | 0,55 |
| High grey level run emphasis_2Davg_ | 0,47 | 0,14 | 0,08 | 0,05 | 0,76 |
| Short run low grey level emphasis_2Davg_ | 0,39 | 0,14 | 0,05 | 0,27 | 0,54 |
| Short run high grey level emphasis_2Davg_ | 0,48 | 0,14 | 0,09 | 0,05 | 0,75 |
| Long run low grey level emphasis_2Davg_ | 0,38 | 0,11 | 0,01 | 0,28 | 0,57 |
| Long run high grey level emphasis_2Davg_ | 0,45 | 0,11 | 0,08 | 0,05 | 0,77 |
| Grey level non uniformity GLRLM_2Davg_ | 0,92 | 0,68 | 0,92 | 0,76 | 0,97 |
| Grey level non uniformity normalised GLRLM_2Davg_ | 0,87 | 1,00 | 0,77 | 0,99 | 0,98 |
| Run length non uniformity_2Davg_ | 1,00 | 1,00 | 1,00 | 1,00 | 1,00 |
| Run length non uniformity normalised_2Davg_ | 0,62 | 0,33 | 0,31 | 0,36 | 0,11 |
| Run percentage_2Davg_ | 0,61 | 0,31 | 0,32 | 0,36 | 0,13 |
| Grey level variance GLRLM_2Davg_ | 0,78 | 0,15 | 0,22 | 0,28 | -0,23 |
| Run length variance_2Davg_ | 0,60 | 0,24 | 0,35 | 0,40 | -0,08 |
| Run entropy_2Davg_ | 0,92 | 0,99 | 0,88 | 0,99 | 0,99 |
| Short runs emphasis_2Dcomb_ | 0,60 | 0,33 | 0,29 | 0,31 | 0,15 |
| Long runs emphasis_2Dcomb_ | 0,60 | 0,29 | 0,32 | 0,34 | 0,15 |
| Low grey level run emphasis_2Dcomb_ | 0,39 | 0,14 | 0,04 | 0,27 | 0,55 |
| High grey level run emphasis_2Dcomb_ | 0,47 | 0,14 | 0,08 | 0,05 | 0,76 |
| Short run low grey level emphasis_2Dcomb_ | 0,39 | 0,14 | 0,05 | 0,27 | 0,54 |
| Short run high grey level emphasis_2Dcomb_ | 0,48 | 0,14 | 0,09 | 0,05 | 0,75 |
| Long run low grey level emphasis_2Dcomb_ | 0,38 | 0,12 | 0,01 | 0,28 | 0,57 |
| Long run high grey level emphasis_2Dcomb_ | 0,45 | 0,11 | 0,08 | 0,05 | 0,77 |
| Grey level non uniformity GLRLM_2Dcomb_ | 0,92 | 0,69 | 0,92 | 0,76 | 0,97 |
| Grey level non uniformity normalised GLRLM_2Dcomb_ | 0,87 | 1,00 | 0,77 | 0,99 | 0,98 |
| Run length non uniformity_2Dcomb_ | 1,00 | 1,00 | 1,00 | 1,00 | 1,00 |
| Run length non uniformity normalised_2Dcomb_ | 0,61 | 0,33 | 0,30 | 0,35 | 0,12 |
| Run percentage_2Dcomb_ | 0,61 | 0,31 | 0,32 | 0,36 | 0,13 |
| Grey level variance GLRLM_2Dcomb_ | 0,78 | 0,15 | 0,22 | 0,28 | -0,23 |
| Run length variance_2Dcomb_ | 0,60 | 0,25 | 0,34 | 0,39 | -0,09 |
| Run entropy_2Dcomb_ | 0,93 | 1,00 | 0,89 | 0,99 | 0,99 |
| Short runs emphasis_3Davg_ | 0,68 | 0,30 | 0,52 | 0,52 | 0,37 |
| Long runs emphasis_3Davg_ | 0,67 | 0,31 | 0,52 | 0,51 | 0,40 |
| Low grey level run emphasis_3Davg_ | 0,37 | 0,58 | 0,07 | 0,71 | 0,72 |
| High grey level run emphasis_3Davg_ | 0,47 | 0,13 | 0,13 | 0,19 | 0,64 |
| Short run low grey level emphasis_3Davg_ | 0,38 | 0,59 | 0,08 | 0,73 | 0,72 |
| Short run high grey level emphasis_3Davg_ | 0,47 | 0,14 | 0,14 | 0,19 | 0,63 |
| Long run low grey level emphasis_3Davg_ | 0,34 | 0,52 | 0,05 | 0,61 | 0,71 |
| Long run high grey level emphasis_3Davg_ | 0,47 | 0,11 | 0,12 | 0,17 | 0,68 |
| Grey level non uniformity GLRLM_3Davg_ | 0,95 | 0,93 | 0,96 | 0,83 | 0,97 |
| Grey level non uniformity normalised GLRLM_3Davg_ | 0,45 | 0,89 | 0,12 | 0,71 | 0,56 |
| Run length non uniformity_3Davg_ | 1,00 | 1,00 | 1,00 | 1,00 | 1,00 |
| Run length non uniformity normalised_3Davg_ | 0,69 | 0,35 | 0,52 | 0,54 | 0,37 |
| Run percentage_3Davg_ | 0,68 | 0,35 | 0,52 | 0,54 | 0,39 |
| Grey level variance GLRLM_3Davg_ | 0,74 | 0,55 | 0,18 | 0,52 | 0,78 |
| Run length variance_3Davg_ | 0,66 | 0,35 | 0,52 | 0,51 | 0,42 |
| Run entropy_3Davg_ | 0,40 | 0,95 | 0,15 | 0,85 | 0,56 |
| Short runs emphasis_3Dcomb_ | 0,68 | 0,34 | 0,52 | 0,54 | 0,37 |
| Long runs emphasis_3Dcomb_ | 0,67 | 0,35 | 0,52 | 0,53 | 0,40 |
| Low grey level run emphasis_3Dcomb_ | 0,37 | 0,58 | 0,07 | 0,71 | 0,72 |
| High grey level run emphasis_3Dcomb_ | 0,47 | 0,13 | 0,13 | 0,19 | 0,64 |
| Short run low grey level emphasis_3Dcomb_ | 0,38 | 0,58 | 0,08 | 0,73 | 0,72 |
| Short run high grey level emphasis_3Dcomb_ | 0,47 | 0,14 | 0,14 | 0,19 | 0,63 |
| Long run low grey level emphasis_3Dcomb_ | 0,34 | 0,52 | 0,05 | 0,61 | 0,71 |
| Long run high grey level emphasis_3Dcomb_ | 0,47 | 0,11 | 0,12 | 0,17 | 0,68 |
| Grey level non uniformity GLRLM_3Dcomb_ | 0,95 | 0,93 | 0,96 | 0,83 | 0,97 |
| Grey level non uniformity normalised GLRLM_3Dcomb_ | 0,45 | 0,89 | 0,12 | 0,71 | 0,56 |
| Run length non uniformity_3Dcomb_ | 1,00 | 1,00 | 1,00 | 1,00 | 1,00 |
| Run length non uniformity normalised_3Dcomb_ | 0,68 | 0,34 | 0,52 | 0,54 | 0,37 |
| Run percentage_3Dcomb_ | 0,68 | 0,35 | 0,52 | 0,54 | 0,39 |
| Grey level variance GLRLM_3Dcomb_ | 0,74 | 0,55 | 0,18 | 0,52 | 0,78 |
| Run length variance_3Dcomb_ | 0,66 | 0,35 | 0,52 | 0,51 | 0,42 |
| Run entropy_3Dcomb_ | 0,43 | 0,95 | 0,13 | 0,86 | 0,60 |
| Small zone emphasis_2D_ | 0,55 | 0,25 | 0,24 | 0,38 | 0,07 |
| Large zone emphasis_2D_ | 0,59 | 0,27 | 0,29 | 0,29 | 0,16 |
| Low grey level zone emphasis_2D_ | 0,39 | 0,16 | 0,06 | 0,10 | 0,58 |
| High grey level zone emphasis_2D_ | 0,47 | 0,14 | 0,08 | 0,00 | 0,76 |
| Small zone low grey level emphasis_2D_ | 0,40 | 0,17 | 0,10 | 0,25 | 0,55 |
| Small zone high grey level emphasis_2D_ | 0,49 | 0,17 | 0,08 | 0,05 | 0,74 |
| Large zone low grey level emphasis_2D_ | 0,34 | 0,06 | -0,02 | 0,30 | 0,68 |
| Large zone high grey level emphasis_2D_ | 0,43 | 0,03 | 0,06 | 0,03 | 0,81 |
| Grey level non uniformity GLSZM_2D_ | 0,94 | 0,70 | 0,95 | 0,00 | 0,98 |
| Grey level non uniformity normalised GLSZM_2D_ | 0,88 | 1,00 | 0,83 | 0,75 | 0,99 |
| Zone size non uniformity_2D_ | 0,98 | 0,97 | 0,96 | 0,94 | 0,99 |
| Zone size non uniformity normalised_2D_ | 0,60 | 0,25 | 0,26 | 0,45 | 0,01 |
| Zone percentage_2D_ | 0,61 | 0,28 | 0,31 | 0,41 | 0,04 |
| Grey level variance GLSZM_2D_ | 0,77 | 0,15 | 0,21 | 0,28 | -0,25 |
| Zone size variance_2D_ | 0,60 | 0,27 | 0,29 | 0,28 | -0,22 |
| Zone size entropy_2D_ | 0,96 | 0,99 | 0,94 | 0,99 | 0,99 |
| Small zone emphasis_3D_ | 0,66 | 0,24 | 0,32 | 0,51 | 0,16 |
| Large zone emphasis_3D_ | 0,51 | 0,45 | 0,46 | 0,39 | 0,48 |
| Low grey level zone emphasis_3D_ | 0,33 | 0,69 | 0,09 | 0,05 | 0,74 |
| High grey level zone emphasis_3D_ | 0,40 | 0,17 | 0,08 | 0,00 | 0,60 |
| Small zone low grey level emphasis_3D_ | 0,36 | 0,55 | 0,09 | 0,63 | 0,79 |
| Small zone high grey level emphasis_3D_ | 0,43 | 0,24 | 0,10 | 0,16 | 0,47 |
| Large zone low grey level emphasis_3D_ | -0,07 | 0,23 | 0,34 | 0,03 | 0,64 |
| Large zone high grey level emphasis_3D_ | 0,77 | 0,15 | 0,25 | -0,04 | 0,88 |
| Grey level non uniformity GLSZM_3D_ | 0,99 | 0,95 | 0,99 | 0,01 | 0,99 |
| Grey level non uniformity normalised GLSZM_3D_ | 0,42 | 0,92 | 0,11 | 0,06 | 0,41 |
| Zone size non uniformity_3D_ | 0,93 | 0,95 | 0,94 | 0,86 | 0,97 |
| Zone size non uniformity normalised_3D_ | 0,67 | 0,24 | 0,33 | 0,52 | 0,16 |
| Zone percentage_3D_ | 0,70 | 0,41 | 0,50 | 0,56 | 0,40 |
| Grey level variance GLSZM_3D_ | 0,70 | 0,55 | 0,15 | 0,52 | 0,66 |
| Zone size variance_3D_ | 0,47 | 0,48 | 0,45 | 0,35 | 0,49 |
| Zone size entropy_3D_ | 0,90 | 0,97 | 0,79 | 0,96 | 0,81 |
| Coarseness_2D_ | 0,46 | 0,55 | 0,47 | 0,28 | 0,95 |
| Contrast_2D_ | 0,68 | 0,83 | 0,14 | 0,85 | 0,30 |
| Busyness_2D_ | 0,33 | 0,48 | 0,57 | 0,78 | 0,66 |
| Complexity_2D_ | 0,51 | 0,22 | 0,22 | -0,01 | 0,34 |
| Texture strength_2D_ | 0,81 | 0,33 | 0,46 | 0,10 | 0,67 |
| Coarseness_3D_ | 0,99 | 0,58 | 0,98 | 0,59 | 1,00 |
| Contrast_3D_ | 0,86 | 0,88 | 0,72 | 0,90 | 0,77 |
| Busyness_3D_ | 0,79 | 0,39 | 0,72 | 0,13 | 0,80 |
| Complexity_3D_ | 0,47 | 0,41 | 0,12 | 0,06 | -0,18 |
| Texture strength_3D_ | 0,96 | 0,75 | 0,76 | 0,70 | 0,98 |

**Table S-2: ICC values for all features; extracted from EARL-compliant reconstruction**

**(OSEM, M256, 4 mm FWHM, CT-based segmentation, FBN discretization)**
